# Clusters of cooperative ion channels enable a membrane-potential-based mechanism for short-term memory

**DOI:** 10.7554/eLife.49974

**Published:** 2020-02-07

**Authors:** Paul Pfeiffer, Alexei V Egorov, Franziska Lorenz, Jan-Hendrik Schleimer, Andreas Draguhn, Susanne Schreiber

**Affiliations:** 1 Institute for Theoretical BiologyHumboldt-Universität zu BerlinBerlinGermany; 2 Bernstein Center for Computational NeuroscienceHumboldt-Universität zu BerlinBerlinGermany; 3 Institute of Physiology and PathophysiologyHeidelberg UniversityHeidelbergGermany; National Heart, Lung and Blood Institute, National Institutes of HealthUnited States; National Heart, Lung and Blood Institute, National Institutes of HealthUnited States

**Keywords:** cooperative ion channels, dynamic clamp, cellular memory, Mouse

## Abstract

Across biological systems, cooperativity between proteins enables fast actions, supra-linear responses, and long-lasting molecular switches. In the nervous system, however, the function of cooperative interactions between voltage-dependent ionic channels remains largely unknown. Based on mathematical modeling, we here demonstrate that clusters of strongly cooperative ion channels can plausibly form bistable conductances. Consequently, clusters are permanently switched on by neuronal spiking, switched off by strong hyperpolarization, and remain in their state for seconds after stimulation. The resulting short-term memory of the membrane potential allows to generate persistent firing when clusters of cooperative channels are present together with non-cooperative spike-generating conductances. Dynamic clamp experiments in rodent cortical neurons confirm that channel cooperativity can robustly induce graded persistent activity – a single-cell based, multistable mnemonic firing mode experimentally observed in several brain regions. We therefore propose that ion channel cooperativity constitutes an efficient cell-intrinsic implementation for short-term memories at the voltage level.

## Introduction

Cooperative molecular interactions are ubiquitous in biology and guide cellular processes from sensing to memory formation (Bray et al., 1998; Burrill and Silver, 2010). They are found not only in the simplest organisms like bacteria, but also in higher organisms including mammals (Stefan and Le Novère, 2013). Evidence increases that also ion channels of excitable membranes in the heart and the nervous system, including the mammalian brain, can exhibit cooperative properties (Choi, 2014; Molina et al., 2006; Grage et al., 2011; Kim et al., 2014; Gianoli et al., 2017; Navedo et al., 2010; Dixon et al., 2015; Moreno et al., 2016; Clatot et al., 2017; Vivas et al., 2017). Nevertheless, the majority of neuron models relies on the independent gating assumption: channels communicate indirectly via the common membrane potential, but do not directly influence each other. Thus, it is an open question how cooperative channels affect the electrical dynamics - and therefore the computations - of a neuron.

Experimentally, ion channel cooperativity has been studied in various channel types with key roles in the nervous system such as potassium (Molina et al., 2006; Kim et al., 2014), sodium (Iwasa et al., 1986; Undrovinas et al., 1992; Clatot et al., 2017), HCN (Dekker and Yellen, 2006) and calcium channels (Dixon et al., 2015; Moreno et al., 2016). However, most studies have focused on the demonstration of cooperative gating in small ensembles of channels and avoided the complex dynamics of the neuron as a whole. An exception is the study of cooperative calcium channels in rodent hippocampal neurons, where optical control of channel coupling has revealed that the spontaneous firing rate rises when channels cooperate (Moreno et al., 2016). With such control of channel interactions, future experiments have the means to test hypothesis on the function of cooperativity.

So far, a few computational studies predict effects of cooperativity. Along these lines, cooperative sodium channels have been suggested to underlie the rapid initiation and low variability of spiking onset in cortical neurons (Naundorf et al., 2006; Huang et al., 2012; for an alternative mechanism see Yu et al., 2008) and mild cooperative interactions in potassium and calcium channels have been shown to modify the steepness of a channel’s activation curve and modulate neural excitability (Zarubin et al., 2012). Furthermore, cardiac alternans, a pathological condition of unstable heart contractions, has been linked to strong degrees of coupling among cooperative calcium channels (Sato et al., 2018). These studies demonstrate that cooperative channels can significantly alter cellular firing properties with a range of effects from advantageous to pathological depending on the interplay with other currents in the cell.

Here, we show in simulations and mathematical analysis that small clusters of cooperative channels with simple and generic activation dynamics can induce a multistability of the membrane potential and demonstrate that this multistability enables a form of cellular memory. Our central observation is that the mutually enhancing nature of cooperative gating favors joint opening and closing of channels, and, more importantly, results in a hysteresis of their gating behavior. We demonstrate that such cooperative channels - when arranged in clusters and located in membranes with ‘normal’ independent conductances that mediate spiking - can induce graded persistent neural activity: spiking that persists after transient suprathreshold depolarization and represents successive inputs with increasing persistent firing rates. Taken together, we propose that cooperativity of a few ion channels is an elegant way to implement a cell-intrinsic memory directly reflected in a cell’s firing. In principle, ion channel cooperativity could thus efficiently complement network-based mechanisms of persistent activation and thereby contribute to decision making (accumulation of evidence) and working memory. Overall, the emergence of memory in a cluster of coupled, yet in isolation memoryless channels suggests a more general design principle: cooperativity serves to dynamically build functionally rich macrochannels from simpler channels.

In the following, we first dissect the mechanism and compare the gating of independent versus cooperative ion channel clusters. We then add clusters of cooperative channels to a simple neuron model with non-cooperative, spike-generating sodium and potassium channels, proving the ability of ion channel cooperativity to mediate graded persistent activity. Finally, we use the dynamic clamp technique to experimentally endow perirhinal cortex neurons with ‘virtual’ cooperative ion channels and show that stable persistent activity can be robustly induced, rendering ion channel cooperativity an efficient and plausible mechanism for cellular voltage memory.

## Results

Although the mechanisms of cooperative interactions among ion channels are still not fully understood on the molecular level, it can be assumed that ion channels need to be in spatial proximity to directly interact and gate in a cooperative manner (Gutkin and Ermentrout, 2006). Matching this assumption, cooperativity is often found in channels that form small clusters (Molina et al., 2006; Choi, 2014; Navedo et al., 2010; Moreno et al., 2016). For our study, we therefore assume that the cooperative channels are distributed in multiple clusters and limit cooperative interactions to channels within the same cluster. In this regard, we deviate from previous theoretical studies, where all-to-all interactions between channels in a membrane have been investigated (Naundorf et al., 2006; Zarubin et al., 2012; Huang et al., 2012). To understand the underlying principles, we begin with a comparison of gating properties in a cluster of cooperative versus non-cooperative ion channels.

### Gating of a cluster of cooperative channels

**Figure 1.**
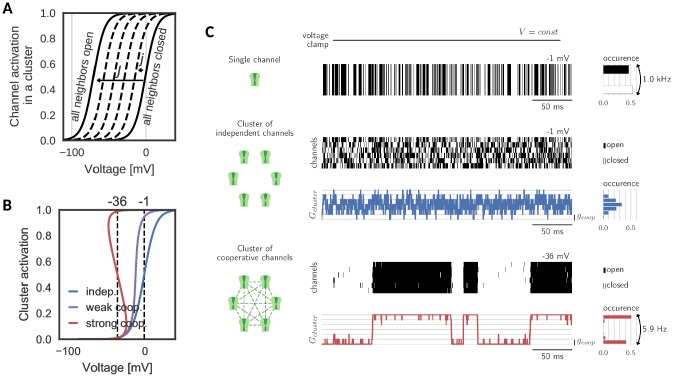
Gating of a cluster of cooperative channels. (**A**) Model of cooperative gating: the activation of a channel in a cluster depends on the state of the surrounding channels. Opening of a neighboring channel leads to a shift of the activation curve by the coupling strength j. When all neighbors are closed, the activation coincides with the one of an isolated channel, whereas when all neighbors are open, the activation undergoes the maximal shift J. (**B**) Cluster activation: cooperative interactions inside a cluster increase the channel activity, so that the activation curve becomes steeper (weak coop.). Above a critical coupling strength, the activation starts to ‘bend over’ (strong coop.); whether a channel is open or closed is determined mostly by the state of its surrounding channels. Activation curve based on a self-consistency relation, for details see Materials and methods. (**C**) *Top*: Simulation of a voltage clamp experiment with an isolated two-state channel shows fast, random switching between the conducting (black) and non-conducting (white) state. Operated at the half-activation potential $V_{0.5}$, the channel spends about half of the time in the open state. *Middle*: In a cluster of independent channels, their asynchronous gating results in a fluctuation of the cluster conductance around half of the total conductance. *Bottom*: Cooperative channels have a strong preference for the state of the surrounding channels. In a cluster, they open and close in synchrony acting like a macrochannel. Its slow switching frequency demonstrates the stability of the open and closed cluster state. As the cooperative channels are more active compared to the independent ones, they are clamped at more hyperpolarized voltages: −1 mV (independent) and 36 mV (cooperative). The details of the jump process simulation are given in the section Materials and methods and parameters are summarized in Table 1.

A cluster is assumed to comprise S voltage-gated channels of the same type, whose individual activation curve is a monotonically increasing, yet saturating function of voltage. To ensure the generality of the analysis, we make no further assumptions on other channel properties like their ionic nature and therefore simply refer to them as ‘channels’. Cooperative interactions are chosen to be enhancing, that is the opening of a channel in the cluster increases the opening probabilities of all other S-1 channels. This increase in opening probability is implemented phenomenologically by shifting the channels’ activation curves to lower voltages for each new opening of a neighbor. The size of the shift j induced by one new opening is a measure of the coupling between channels in the cluster (Naundorf et al., 2006; Zarubin et al., 2012; Huang et al., 2012). An important determinant for the cluster dynamics is the maximal shift J=(S-1)⁢j, which a channel experiences, when all neighbors are in the open state (Figure 1A). Variation of this interaction strength from independent (J = 0 mV) to strongly cooperative (J = 70 mV) yields the activation curve of a cluster of S channels. Clearly, an isolated channel and channels in an independent ensemble have the same activation curve. Mild cooperative interactions, however, increase the channel activity and make the activation curve steeper. Beyond a critical coupling, the activation curve ‘bends over’, which characterizes the regime of strong cooperativity (Figure 1B).

**Table 1.** Cluster and cooperative channel parameters. N: number of clusters, S: cluster size, J: overall coupling, j: channel-channel coupling, $V_{0.5}$: half activation, k: width of the channel activation, τ: time constant of the channel with maximum at attained at $V_{m}$ and width σ, $g_{c\,o\,o\,p}$: single channel conductance and $E_{c\,o\,o\,p}$: reversal potential.

Figure	N	S	J	j	$V_{0.5}$	k	τ	$V_{m}$	σ	g_coop_	E_coop_
			mV	mV	mV	mV	ms	mV	mV	pS	mV
Figure 1 (independent)	1	6	0	0	−1	15	0.5	−1	30	-	-
Figure 1 (weak coop.)	1	6	22.5	4.5	−1	15	0.5	−1	30	-	-
Figure 1 (strong coop.)	1	6	70	14	−1	15	0.5	−1	30	-	-
Figure 2 (independent)	1	6	0	0	−1	15	0.5	−1	30	-	-
Figure 2 (cooperative)	1	6	100	20	−1	15	0.5	−1	30	-	-
Figure 3	1	5	100	25	−1	15	0.5	−1	30	-	-
Figure 4	100	8	80	11.4	−30	10	120	−30	20	2.5	100
Figure 5	100	8	80	11.4	−30	10	120	−30	20	2.5	100
Figure 6c	90	8	101.5	14.5	−10	15	100	−10	30	1	100
Figure 3—figure supplement 1	1	5	100	25	−1	15	0.5	−1	30	-	-
Figure 4—figure supplement 1A	100	8	80	11.4	−30	10	120	−30	20	2.5	100
Figure 4—figure supplement 1B	100	8	80	11.4	−30	10	120	−30	20	10	−100
Figure 4—figure supplement 2	100	8	80	11.4	−30	10	120	−30	20	2.5	100
Figure 5—figure supplement 1A,B	100	8	80	11.4	−30	10	10–240	−30	20	2.5	100
Figure 5—figure supplement 1C,D	100	8	80	11.4	−30	10	120	−30	20	0.5–10	100
Figure 6—figure supplement 1 (independent channels, red)	90	8	0	0	−10	15	100	−10	30	1	100
Figure 6—figure supplement 1 (cooperative channels, black)	90	8	101.5	14.5	−10	15	100	−10	30	1	100

Simulating a simple voltage clamp experiment in a membrane patch containing a cluster of six channels demonstrates fundamental differences between the gating of independent and cooperative clusters: The conductance dynamics of a cluster of independent channels is identical to the sum of its individual, independent components. Therefore, the single channel opening probability dictates the most probable cluster state (defined by the channel activation curve and the voltage) and the cluster conductance fluctuates around this state following a binomial distribution (Figure 1C, top). At the half-activation voltage, the cluster average is at half of its total conductance. Fluctuations in the total cluster conductance are relatively fast (due to channel gating time constants of ≈ 1 ms).

The picture changes when ion channel cooperativity is introduced. Assuming strong interactions, all channels in a cluster gate open and close quasi-simultaneously (at a voltage level within the regime where the activation curve ‘bends over’). Intuitively, this coordination within a cluster can be easily understood: the opening of ion channels strongly enhances the probability of their neighbors to open. Similarly, the closing of channels reduces the probability of their neighbors to remain open, so that gating dynamics largely synchronize. The distribution of total cluster conductance now deviates from the unimodal, binomial distribution of clusters with independent channels: it becomes bimodal reflecting the alternation between a fully closed and a fully open cluster state (Figure 1C, bottom). Note that the bistability is accompanied by a novel slow time scale; the cluster switches between the all-open or all-closed states at a drastically reduced rate (6 Hz) compared to its constituting channels when independent (1000 Hz). Effectively, the bistability and the slow switching let the cluster of cooperative channels appear as one slow macrochannel (Blunck et al., 1998).

### Hysteresis and bistable gating

**Figure 2.**
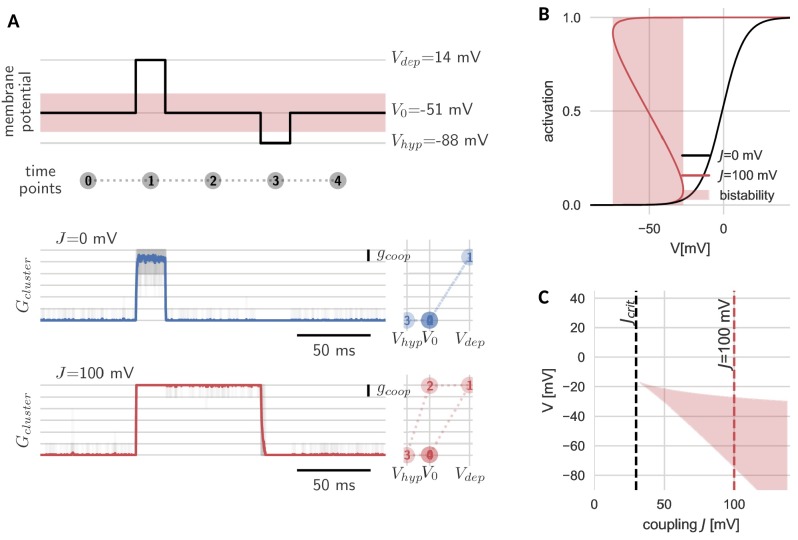
Hysteresis and bistability of a cluster of cooperative channels. (**A**) Probing an initially closed cluster (time point 0), both independent (blue) and cooperative (red) channels open in response to a depolarizing voltage pulse (1). However, returning to the base line voltage (2), independent channels rapidly close again, whereas a cluster of cooperative channels ‘sticks’ to the open configuration. Only a strong hyperpolarizing pulse (3) can close the cooperative channels and restore the initial condition (4). Tracking membrane potential versus conductance during the pulse protocol (right panels), the hysteresis in gating for the cooperative channels becomes apparent; whether the cluster is open or closed at baseline voltage depends on the previous voltage pulse. The transparent lines correspond to 20 repetitions of the pulse protocol and the solid lines are the respective means. (**B**) Strong coupling among channels changes the activation in a cluster (red) with respect to an isolated channel (black) and induces a voltage regime of bistability (red shaded). (**C**) Clusters only become bistable beyond a critical coupling strength (black dashed) and for increasing coupling, the bistable range extends to lower voltages. Cluster and channel parameters are summarized in Table 1.

The new, slow timescale also changes the response of the cluster to a pulsatile stimulation protocol. During a sufficiently strong depolarizing pulse, all channels in both the cooperative and independent cluster open. Once the pulse is over and voltage returns to baseline, the cluster of independent channels follows and channels swiftly return to the closed state. In the case of the cooperative cluster, however, channels in the cluster remain open, despite the return of the clamped voltage value to its original value before the pulse onset. The strong channel interactions introduce what we would like to term ‘stickiness’: the cluster ‘sticks’ to its new open state (even when voltage levels are back to ‘normal’) and can only be released from this state by a sufficiently strong hyperpolarization (Figure 2A). In other words, cooperative gating induces hysteresis and hence allows channels in a cluster, which are fast switching in isolation, to represent (i.e. ‘remember’) voltage values that occurred multiple hundred milliseconds ago.

The hysteresis in gating arises from the bistability of the cluster. Before the depolarizing pulse, the channels in the cluster are closed, which is a stable cluster state, because the single channel is not activated at this membrane potential and there is no facilitation between closed channels. During the pulse, channels open, and this open cluster configuration is stable at the baseline voltage, because the strong coupling allows the channels to secure each other in the open state. Only when the voltage is lowered further, it overcomes the mutual facilitation and the channels close again.

The bistability reflects those effective cluster activation curves that ‘bend over’ (Figure 2B). Consequently, cluster size, channel coupling and the shape of the isolated channel’s activation curve determine the voltage range of bistability. Both an increase in channel coupling j and cluster size S can lead to a stronger overall cooperativity strength J, which is the product of the two. Bistability arises from a critical overall cooperativity strength $J_{c\,r\,i\,t}$, which depends on the shape of the single channel activation. With increasing J, the bistable range broadens and extends to more hyperpolarized voltages (Figure 2C). Correspondingly, for a cluster with more channels and/or strong coupling, which both result in a larger J, the voltage pulse to close the cluster has to be stronger in order to leave the bistable range.

Due to their self-excitatory effect, channels in a cooperative cluster can be compared to neurons in a recurrently connected network: if the external input is low, the neurons are silent, but during a short stimulation they switch to a very active state, which they sustain after the stimulation by recurrent self-excitation. Such networks have been proposed to underlie the short-term storage of stimuli in working memory (Durstewitz et al., 2000). This poses the question, whether in a similar way, a cluster of cooperative channels can act as a memory unit for a single neuron. In order to address this question, we first need to better understand the emerging slow timescale of switches between cluster states in the cooperative regime.

#### Prolonged lifetimes of the open and closed cluster state

**Figure 3.**
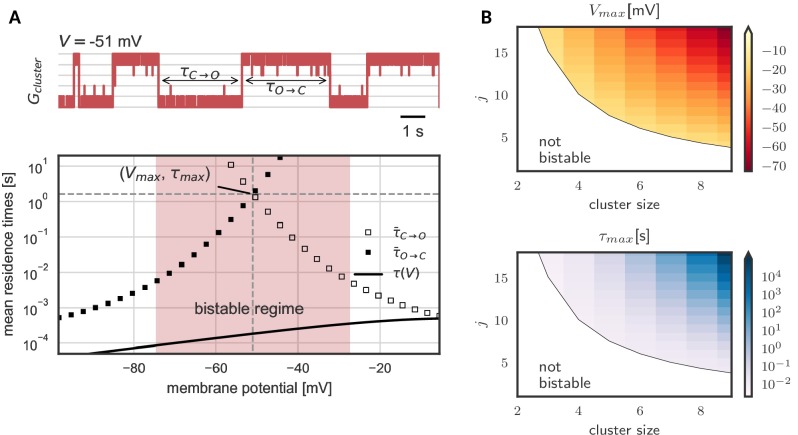
Prolonged life times of cluster states in the bistable regime. (**A**) *Top*: Voltage-clamped in the bistable regime, a cluster of five cooperative channels can stay in the open or closed state for multiple seconds until it spontaneously switches. The noise source are spontaneous single channel gating events, visible as the fast fluctuations around the stable states. *Bottom*: Entering the bistable regime, the residence times in the open and closed state increase over multiple orders of magnitude. The point of maximal stability $\left(V_{m\,a\,x},\tau_{m\,a\,x}\right)$ is located at the center; here, a cluster is expected to stay open or closed for seconds, exceeding by far the single channel time constant τ⁢(V) (black line). Mean life times are derived from a first passage analysis, see Materials and methods. (**B**) Stability of the open and closed cluster configuration depend on the cluster size and the intra channel coupling j. Larger clusters with stronger intra-channel coupling can be stable for multiple seconds and their bistable range moves to more hyperpolarized voltages. Note that small clusters with weak coupling are not bistable (white region). Cluster and channel parameters are summarized in Table 1.

As we have seen, hysteresis allows clusters to act as a voltage memory. After high voltages, they remain open and after low voltages, they remain closed. Eventually, however, a cluster spontaneously switches its state - in a quasi-synchronous fashion all channels open or close (Figure 3A). These switches originate in channel noise, stochastic gating of individual channels. If multiple channels coincide in their spontaneous gating, their neighbors quickly follow. Both opening and closing of channels spread by cooperative coupling; in a spiral of facilitation build-up or, reversely, in a spiral of facilitation loss (Figure 3—figure supplement 1). These gating avalanches, however, cannot be triggered by single or few channels, so that the life times of a cluster can be orders of magnitude longer than those of the single channel.

Figure 3 shows the lifetimes of the open and closed states - exemplary in a cluster with five channels - as a function of voltage. These lifetimes are defined as the time $\tau_{O\to C}$ it takes, once all channels are open until all channels are closed again, and vice versa for $\tau_{C\to O}$. In the center of the bistable range, both open and closed cluster state are very robust against channel fluctuations with mean residence times in the range of seconds (Figure 3A). However, at the verges of the bistable range, channel noise corrupts the stability. At low voltages, an open cluster switches back to the closed state within ≈ 10 ms, whereas at high voltages, within in the same time, a closed cluster spontaneously opens. Thus, for a persistent representation of recent voltage history, the middle voltage range offers the longest memory lifetimes: here both the probability to lose an open cluster state and to lose a closed cluster state are low - a prerequisite for encoding of previous voltages in a persisting cluster state (Figure 3A). The lifetimes in this voltage range define the memory timescale $\tau_{m\,a\,x}$ of the cluster, that is the time during which a cluster of cooperative ion channels can act as a reliable memory device. For completeness, Figure 3B shows the numerically derived center of the bistable range $V_{m\,a\,x}$ and the memory timescale $\tau_{m\,a\,x}$ for clusters of different sizes and with different channel-channel coupling j. We find that both, larger cluster size and stronger channel coupling, favor bistability. Although quantitative data on cooperative interactions is scarce, we note that for a cluster of eight channels coupled with strength j = 17 mV, the mid-voltage of the bistable range decreases to −60 mV and the average lifetime of the open and closed state exceeds hundreds of seconds, rending this paradigm suitable for persistent activation in the context of short-term memory.

In general, our analysis shows that, as expected, larger and more strongly coupled clusters have a more hyperpolarized operation voltage and longer life times. Even for clusters with the same maximal shift J (that is hyperboles in Figure 3B), we observe a pronounced increase in lifetime with cluster size. In other words, large clusters of weakly coupled channels tend to be more stable than small clusters of strongly coupled ones. This observation is explained by a reduction of effective channel noise when more channels are added to a cluster.

**Figure 3—figure supplement 1.**
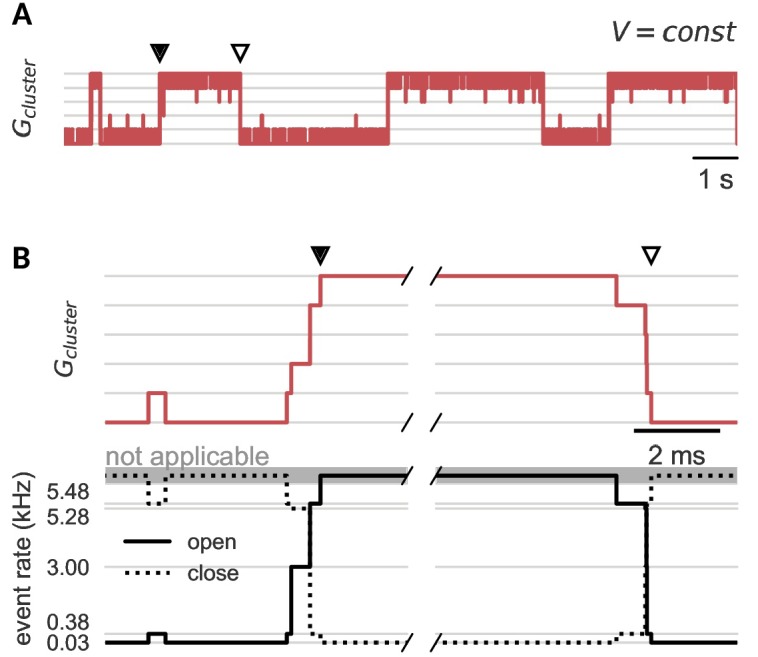
Channel noise can open and close a cluster. (**A**) Clamped at a fixed voltage in the bistable regime, a cluster of cooperative channels slowly switches between the open and closed state. In both cases, when a cluster opens (black dot) or closes (white dot), this quasi-synchronous gating of all channels is triggered by channel noise and amplified by cooperativity. (**B**) Zoom on the exemplary opening (left) and closing (right) events in *A*. The cooperative interactions spread single channel switches by changing the rates for the next opening (solid) or closing (dashed) event. Either channels open and facilitate further openings or channels close and decrease the facilitation of their neighbors. Note that the cluster is stable against single channel gating events; when only one channel opens, the increased opening rate (0.03 kHz to 0.38 kHz) is still ten-fold smaller than the closing rate (5.48 kHz). Cluster parameters are summarized in Table 1.

### Clusters as cellular memory units can mediate persistent spiking

The analysis so far showed that clusters of cooperative ion channels can implement a memory of recent voltage levels in their opening state. Next, we demonstrate how this property - when exhibited by small clusters of channels not even directly involved in the generation of spikes - can lead to persistent neuronal activity that does not require further network input. Interestingly, we find that clusters of cooperative channels can even solve the computationally harder problem required for a graded form of persistent activity. In this graded form, the neuron can signal previous input strength and input duration in the frequency of long-term stable firing. Hence, we propose that ion channel cooperativity offers a generic mechanism for cellular memory.

#### Neural signaling events lead to controllable, persistent cluster switches

To this end, we extend a Hodgkin-Huxley type neuron model including (independent) sodium and potassium channels by a set of 100 small clusters of cooperative channels with the generic activation kinetics described above (for details on the neuron model, see Materials and methods). On the ionic nature of the latter, we assume for the moment that they conduct a depolarizing current like for example calcium channels, which are a potential candidate (Moreno et al., 2016). For the small number of cooperative channels, we simulate their stochastic gating, whereas for the other ionic currents, we use a deterministic simulation.

**Figure 4.**
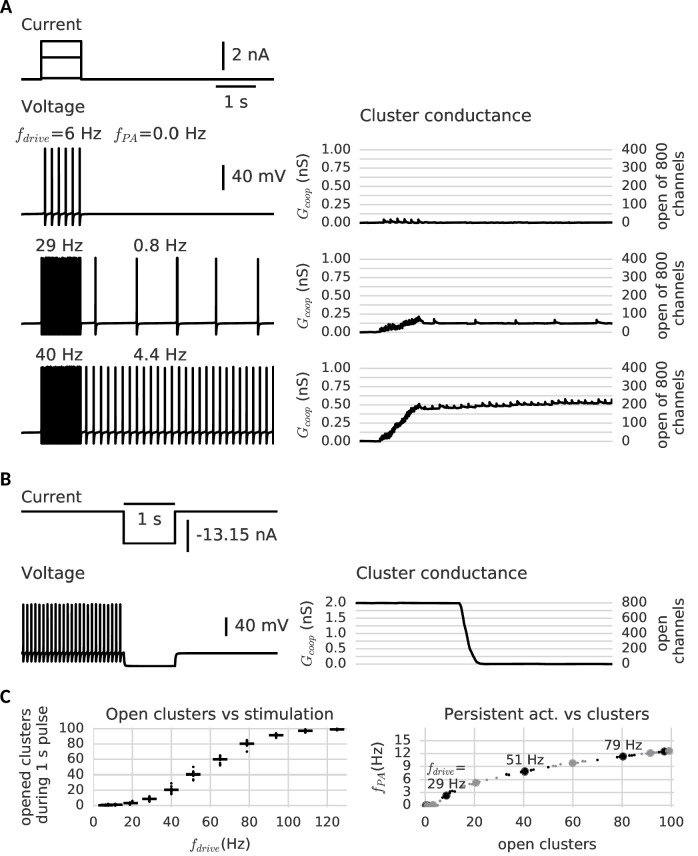
Clusters of cooperative channels mediate persistent activity at different levels in a model neuron. (**A**) Current stimulation with different amplitudes to test the response of clusters to different firing rates. *Left*: Voltage traces demonstrating stimulated firing at increasing frequencies. Whereas the neuron returns to rest after a low amplitude stimulation, after a stronger drive the neuron continues to spike at a stable frequency $f_{P\,A}$, which increases with the frequency of the stimulated firing $f_{d\,r\,i\,v\,e}$. *Right*: Persistent activity is mediated by the conductance of the clusters, which builds up during high-frequency spiking and remains stable during low-frequency spiking and at rest. (**B**) A strong hyperpolarizing step closes the clusters and stops persistent firing. (**C**) *Left*: The cooperative clusters track the input strength; more clusters open when the cell fires at higher frequencies. Firing below 20 Hz, however, does not open clusters. The number of open clusters can differ across trials (black dots) because of stochastic channel gating. *Right*: Persistent activity increases with the number of open clusters, thereby allowing the neuron to represent the input strength after the stimulus has ended, for example 3 Hz after 29 Hz firing and 9 Hz after 51 Hz firing. Big dots denote the mean for each stimulation strength, small dots the individual trials. The simulation procedure is described in Materials and methods and parameters are summarized in Table 1.

Stimulating the model cell with transient input pulses elicits spiking at a rate that increases with the stimulus amplitude (Figure 4). Correspondingly, the stimulation amplitude determines the response of the cooperative channels: At low input amplitudes (and consequently low ensuing firing rates), few channels in the clusters open transiently, but close again rapidly (Figure 4A, top trace). When the pulse is over, the cell falls silent and does not generate spikes. In contrast, if the firing rate during the pulse is large enough, in a subset of clusters all channels open. Moreover, in agreement with our above observations, these clusters now remain open when the input pulse is over (Figure 4A, middle and bottom traces). This additional depolarizing conductance suffices to keep the cell in a firing modus and the model neuron exhibits firing activity that persists beyond the presentation of the stimulus pulse.

**Figure 4—figure supplement 1.**
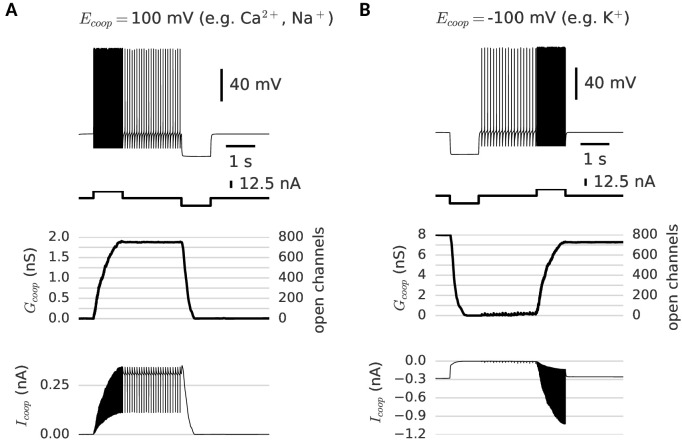
Depending on their reversal potential, clusters of cooperative channels can mediate de- or hyperpolarization-activated persistent activity. (**A**) Depolarization-activated persistent activity. After stimulated spiking, the neuron continues to spike, because of the depolarizing current ($E_{c\,o\,o\,p}$=100 mV) through the persistently open clusters (bottom trace). (**B**) Hyperpolarization-activated persistent activity. A very distinct mnemonic firing behavior is possible with clusters of cooperative channels that have the same dynamics as the ones in *A*, but conduct a hyperpolarizing current ($E_{c\,o\,o\,p}$=-100mV). When such clusters are open initially, they provide a standing leak current that prevents the neuron from firing (bottom trace). A strong hyperpolarizing pulse persistently closes the clusters and thereby activates low-frequency spiking. Strong stimulated spiking reopens the clusters and hence silences the neuron. For a summary of cluster parameters, see Table 1.

**Figure 4—figure supplement 2.**
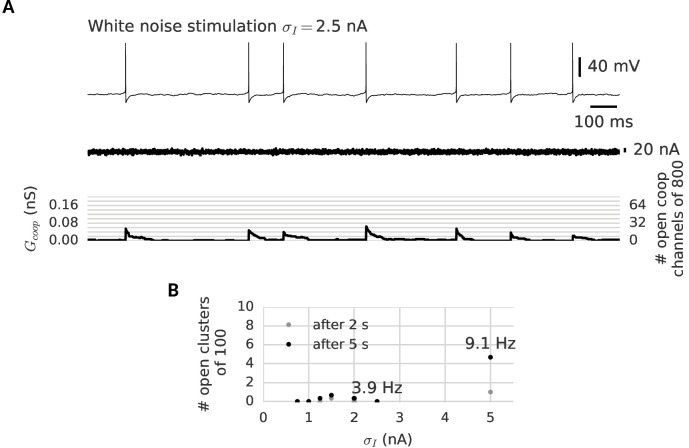
Clusters are robust against noise in the membrane potential. (**A**) A neuron is stimulated for 2 s with a white noise current (middle) to induce membrane potential fluctuations and spontaneous spikes (top). Despite the noisy membrane potential, the clusters remain in their closed state (bottom). Even spontaneous spikes only open a few channels transiently in each cluster and cannot switch a whole cluster persistently to the open state (**B**) The clusters remain closed after exposure to 5 s of noise stimulation. Only an unphysiologically high noise level leads to the opening a few clusters by spontaneous firing at around 10 Hz (see indicated firing frequencies). For details on the white noise stimulation see Materials and methods. There are 100 clusters of eight cooperative channels each. Further parameters are summarized in Table 1.

Furthermore, the larger the amplitude of the stimulating pulse, the larger the persistent firing rate following the pulse (owing to the persistent opening of more clusters). As every cluster of cooperative channels has a stable open and closed configuration, the population conductance across all clusters allows for a quasi-continuous range of persistent currents. When all cluster are open, the maximal current is reached and the persistent firing rate saturates (≈ 15 Hz). According to the gating properties of cooperative channel clusters, sufficiently hyperpolarizing pulses should be able to close clusters and hence ‘reset’ the memory of the previous pulse by annihilation of the persistent activity. Indeed, Figure 4B shows that a hyperpolarizing pulse cancels persistent activity.

In order to ensure maximal stability when there are no inputs to the neuron, the center of the cluster bistability (−70 mV) coincides with the resting potential (−67 mV). The limited extent of the bistable range from around −90 mV to −50 mV allows the clusters to react to spikes or strong inhibition. For the occurrence of graded levels of persistent activity, we choose channels that do not open instantaneously during a spike. Otherwise, a single spike would open all clusters at once. In contrast, for slower channels with a time constant exceeding the width of a single action potential (>10 ms), only a small ratio of the closed channels opens per spike, so that the conductance gradually increases. The channels considered here are even more inert (≈ 100 ms), comparable to slow adaptation currents that require multiple spikes to fully develop (Benda and Herz, 2003). Similarly, clusters of inert channels require multiple spikes in fast succession to switch to the open state (minimum of ≈ 20 Hz, see Figure 4C). In this way, persistent activity at low frequencies cannot open further clusters and remains stable. Similarly, we find that the slow channels make the clusters robust against fluctuation-driven firing (Schreiber et al., 2009) - action potentials triggered by noise in the membrane potential (see Figure 4—figure supplement 2). Finally, the cell is slightly depolarized, so that it spikes in the absence of a stimulus when several clusters are open (see Figure 4C). Even when there are too few open clusters to induce spiking, they still increase the membrane potential and leave a form of ‘silent’ memory (not shown).

Interestingly, the bistable clusters also support an ‘inverse’ form of neuronal memory - persistent activity activated by hyperpolarization and silenced by strong depolarization. For this other form, the cooperative channel dynamics are the same, but they are required to conduct a hyperpolarizing current like for example potassium channels. Then, open clusters act as an additional standing leak current and prevent the cell from firing. Permanently closing the clusters by a hyperpolarizing pulse relieves the cell from this additional leak and allows persistent firing. However, when the cell is stimulated to spike strongly, the clusters return to the open state, restore the standing leak and again silence the cell (see Figure 4—figure supplement 1). Similarly to depolarization-activated persistent activity, the memory relies on the cooperative dynamics of the channels, but how the neuron makes use of this memory changes with their ionic nature.

#### Cooperative channels mediate graded persistent activity

Graded persistent activity is an intriguing, cell-intrinsic feature of some neurons that has been reported in vitro under carbachol in the entorhinal cortex, the perirhinal cortex and the amygdala (Egorov et al., 2002; Navaroli et al., 2012; Egorov et al., 2006). These neurons fire at high frequency in response to depolarizing current pulses and continue to fire stably when the stimulus is over. The persistent firing rate is lower than the one during the pulse and increases with each new pulse presented. Hyperpolarizing pulses lead to graded decreases in firing rate and finally end the persistent activity, seemingly resetting the cell. While other mechanisms reproducing this firing mode have been suggested in the literature (Loewenstein and Sompolinsky, 2003; Fransén et al., 2006), we here add a novel candidate and show that cooperative clusters allow to capture essential properties of graded persistent activity.

**Figure 5.**
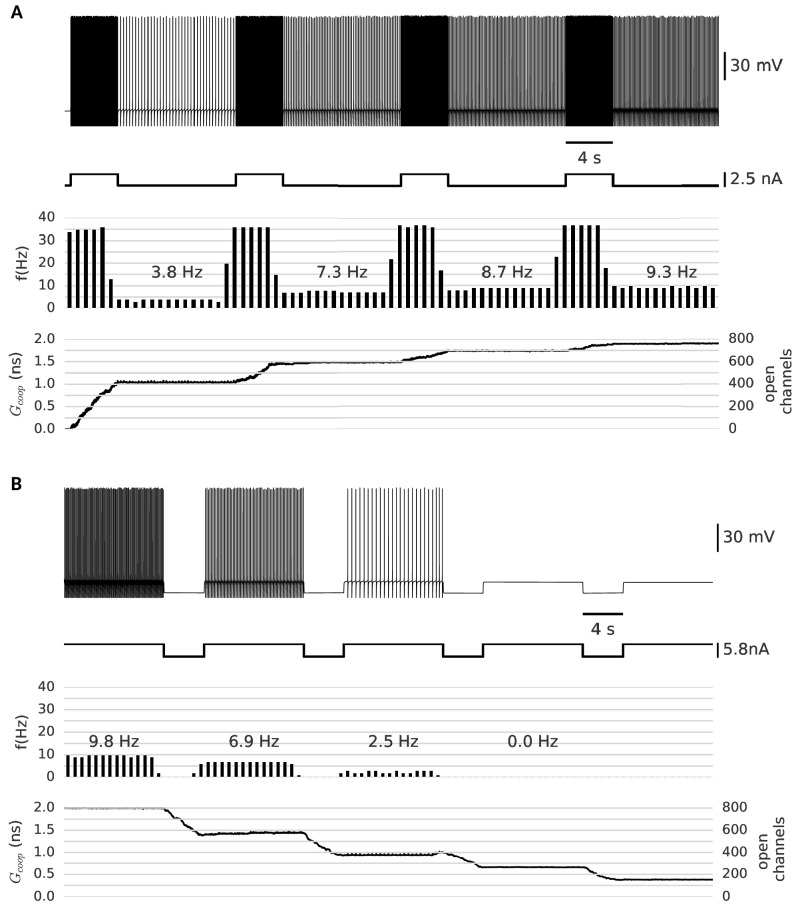
Clusters of strongly cooperative ion channels mediate graded persistent activity in a simple neuron model. (**A**) Repeated stimulation drives fast spiking in the neuron, which is followed by self-sustained, stable low-frequency activity at increasing rates (top). With each pulse, the driven, fast spiking switches more clusters to the open state and adds further stable conductances (bottom). The built-up conductance allows a persistent current to flow and sustains activity beyond stimulation. (**B**) In the same manner, strong hyperpolarization closes the clusters and allows to reduce the frequency of persistent activity until finally the cells stops firing. The simulation procedure is described in Materials and methods and parameters are summarized in Table 1.

In analogy to the experimental paradigm used by Egorov et al. (2002), our neuron model was subjected to a series of depolarizing current pulses. Figure 5A shows the persistent firing after the first pulse as well as the increase of the frequency after each pulse. The persistent frequency was stable for at least one minute (data not shown). The role of the clusters as the underlying memory variable becomes apparent in the step-like evolution of their conductance, which increases during the pulses and stays constant in between. In this simulation, we observe four distinct stable persistent frequencies (from about 3 Hz to 10 Hz). At 10 Hz, further depolarizing pulses fail to increase the frequency of persistent firing. This saturation is reached, when all clusters have been switched to the open state. In addition, we confirmed that persistent firing can be gradually turned off by hyperpolarizing pulses (Figure 5B).

**Figure 5—figure supplement 1.**
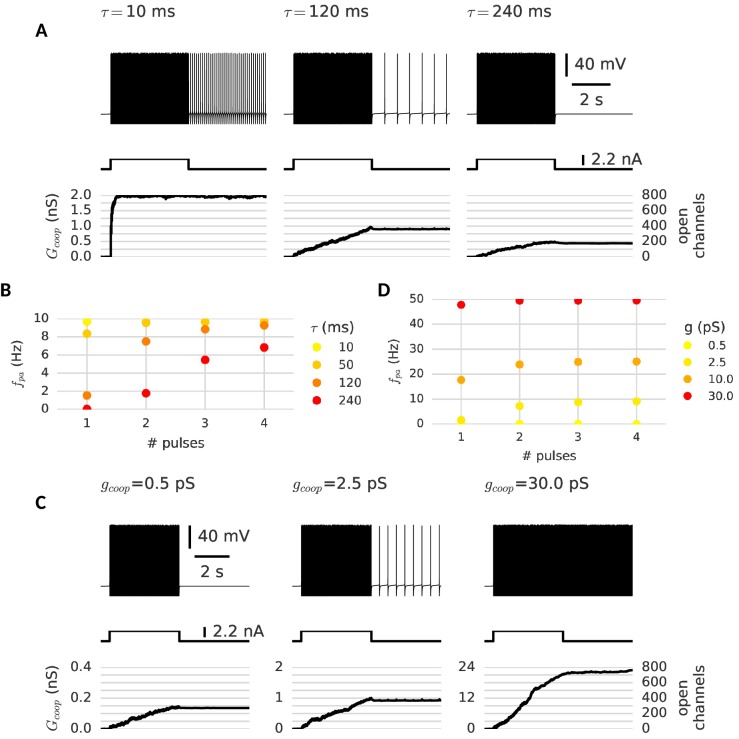
Memory dynamics with different properties of cooperative channels. The channel’s time constant and their conductance change the memory dynamics of the neuron probed with the multi-pulse protocol for graded persistent activity. (**A**) Variation of the time constant: When channels are fast (left, τ = 10 ms), the neuron is already maximally active after the first pulse - a few spikes suffice to open all clusters and there is only one level of persistent activity. Slower channels allow for a graded opening of channels and intermediate levels of persistent activity (middle, τ = 120 ms). If the channels are too slow and too few clusters open during the first pulse, the neuron remains silent (right, τ = 240 ms). (**B**) Frequency of persistent activity after each pulse for different time constants. (**C**) Variation of the channel conductance: When channels are low conducting, the persistent current remains subthreshold and is thus insufficient to drive persistent activity (left). At an intermediate conductance, the clusters mediate low frequency persistent activity (middle). When the provided conductance is too large, the high frequency persistent activity can open further clusters and is no longer stable. (**D**) Frequency of persistent activity after each pulse for different channel conductances. Other cluster parameters are summarized in Table 1.

We further investigated which other channel properties - next to strong cooperativity - are essential for the graded nature of persistent activity (Figure 5—figure supplement 1). We concentrated on the time constant and the conductance of the channels. Fast channels, as discussed in the previous section, are less suited than slow channels. The slow channels ensure that clusters gradually open during stimulated fast spiking and halt during slow spiking, so that the persistent firing remains stable. The conductance of the channels has to be at an appropriate intermediate level: sufficient to drive the neuron over threshold, but limited to provide persistent firing frequencies of maximal 10–15 Hz. Otherwise, faster spiking can open further clusters and the persistent activity increases, so that it is no longer stable.

Concluding, a small set of cooperative clusters of ion channels - when combined with ‘normal’, non-cooperative potassium and sodium channels (responsible for the generation of action potentials) - can reproduce the computationally relevant feature of graded persistent activity.

#### Cooperative channels induce a switch to mnenomic firing in perirhinal cortex neurons

**Figure 6.**
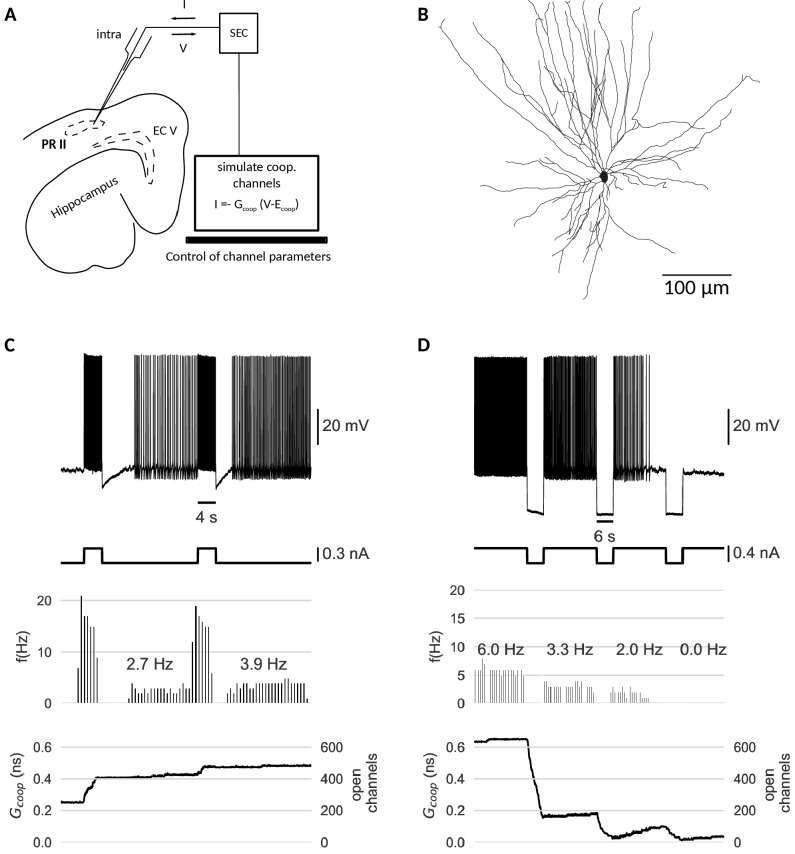
Dynamic clamp experiment. (**A**) Intracellular recording of a perirhinal cortex neuron in dynamic clamp mode. The computer simulates the state of clusters of cooperative channels given the measured membrane potentials and emulates the effect of their conductance to the neuron’s activity by inserting a corresponding current. (**B**) Neurolucida drawing of the recorded neuron. (**C**) Graded persistent activity of the recorded neuron mediated by the multi stable conductance of the clusters of cooperative channels. (**D**) Hyperpolarization brings the cell back to rest via intermediate levels of persistent firing. In control recordings with identical, yet- independent channels, no persistent firing was observed (Figure 6—figure supplement 1). Slice preparation, electrophysiological setup and the dynamic clamp emulation of the clusters are described in Material and methods. Cluster parameters are summarized in Table 1.

Last but not least, we test the cooperativity mechanism experimentally. Our simulations indicate that clusters of cooperative ion channels can enable the mnemonic firing mode of graded persistent activity in a simple point neuron. In biological neurons, however, other ionic currents, morphological effects or noise could interfere with the action of the cooperative channels and undermine their role as memory units. Thus, we employed the dynamic clamp technique (Sharp et al., 1993; Prinz et al., 2004) to equip perirhinal cortex (PR) neurons in-vitro with the same cooperative clusters used in our models, acting as ‘virtual channels’ in real cells (Figure 6). PR neurons have been shown to exhibit graded persistent activity under activation of muscarinic acetylcholine receptors (Navaroli et al., 2012). Consequently, we applied no carbachol, so that any form of persistent activity observed in the recorded PR neurons stems from the clusters of cooperative channels introduced via dynamic clamp. Accordingly, all recordings were performed in the presence of a synaptic blocker to exclude network contributions. For details of experiments, see Materials and methods.

**Figure 6—figure supplement 1.**
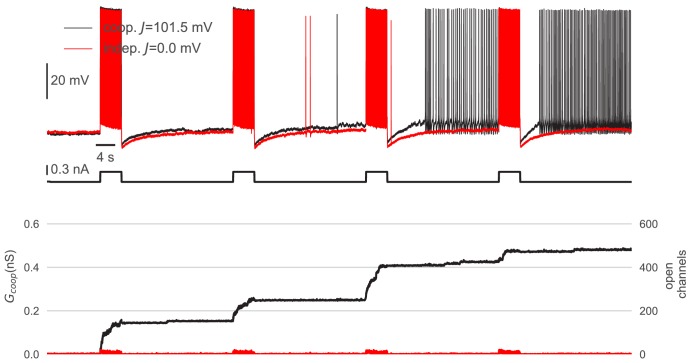
Cooperative interactions are necessary to mediate graded persistent activity. Control experiment to demonstrate the requirement of cooperative channel interactions for graded persistent activity in a perirhinal cortex neuron. *Top*: When the channels gated independently (red, J=0 mV), the neuron only fired during the stimulating pulses and otherwise remained silent. When, in contrast, the channels cooperate strongly (black, J=101.5 mV), the cell produced graded persistent activity (this trace is the full trace of the extract shown in Figure 6). *Bottom*: The independent channels reacted to spiking activity only in a transient way and remained closed after the stimulus. In contrast, cooperative channels opened and facilitated their neighbors to open as well. In this way, spiking could open whole clusters, which remained open after the stimulus and drove persistent activity. A detailed description of the dynamic clamp experiment is given in Material and methods. Cluster parameters are summarized in Table 1.

The dynamic clamp technique allowed us to control the cluster size, channel interactions and channel kinetics of the artificial conductances during the experiment. Like in the model neuron simulations, we adapted the cluster parameters so that the bistability range fitted to the properties of the recorded cell.The resting potential determined the center of the bistable range, whereas the spike shape set boundaries for the bistable range. Voltage values during afterhyperpolarization lied above the lower boundary (to prevent reset by a spike’s afterhyperpolarization), but the upstroke of the action potential exceeded the upper boundary of the bistable range.

Stable recordings with cooperative channel dynamics added via dynamic clamp were obtained for three PR neurons. Under cooperative conditions, all cells exhibited persistent activity in response to current stimulation with pulses (Figure 6C). Persisting activity following pulses was graded, that is the persistent firing rate increased with each presented pulse. As in the mathematical model, hyperpolarizing pulses lowered the rate of persistent activity and when applied multiple times, silenced it (Figure 6D). In the control conditions (identical, yet non-cooperative channel clusters), no persistent firing could be evoked (Figure 6—figure supplement 1).

Periods of stimulation were followed by a slow afterhyperpolarization (sAHP) in both control and cooperative conditions, which for paradigms with cooperative gating generated a 3–4 s lasting refractory period between the stimulation and the self-sustained firing. Interestingly (and in agreement with mathematical modelling), the persistent activity ‘unfolded’ despite these intrinsically generated dynamics. We note that such sAHP were not expected in the original recordings of carbachol-induced persistent activity (Egorov et al., 2002), as carbachol blocks sAHPs.

Taken together, the experimental data support the hypothesis that cooperative ion channels can mediate cellular persistent activity. The persistent dynamics are very robust and could be easily evoked without further knowledge of the intrinsic properties of the recorded cells, such as the multitude of other ionic currents, complex cell morphology, and the presence of channel noise.

## Discussion

Despite a large body of evidence for cooperative interactions between channels pivotal to the nervous system (Iwasa et al., 1986; Dekker and Yellen, 2006; Kim et al., 2014; Choi, 2014; Dixon et al., 2015; Moreno et al., 2016; Clatot et al., 2017), it is unknown which function coupled channels have for neural dynamics and computation. Based on a mathematical analysis, we show in this study that a cluster of cooperative channels can gate with hysteresis and find, in both simulations and experiments, that multiple such clusters embedded in the membrane of a neuron mediate mnemonic firing like persistent activity. Therefore, we suggest that ion channel cooperativity might serve as a cell-intrinsic memory mechanism at the voltage level.

From synaptic learning, adaption of the immune system to epigenetics, many biological memory systems are founded on strong positive autofeedback (Lisman, 1985; Burrill and Silver, 2010). Feedback loops are ubiquitous in biology (Thomas and D’Ari, 1990); they amplify signals as in calcium-induced calcium release and enhance sensitivity in controlling protein function by multisite phosphorylation. In particular, strong feedback enables bistability and memory as in networks of recurrently connected neurons (Amit, 1990; Durstewitz et al., 2000). Here, we exploit the same principles for voltage-gated ion channels, where cooperativity can act as a strong auto-feedback and enables clusters of channels to act as bistable macrochannels with a memory of previous voltage levels.

### Bistability and memory of a cooperative channel cluster

Our gating analysis shows that clusters only become bistable, when channels cooperate strongly. We suggest two criteria to detect this strong coupling regime: a bimodal distribution of cluster conductance states and a hysteresis in gating. Along these lines, experimental evidence for the strong coupling regime comes from cooperative calcium channels in the heart (Navedo et al., 2010). Although these channels couple weakly for the majority of clusters, a few clusters are bistable, switching between the all open and the all closed state, which implies the presence of strong coupling.

Our study of channel noise confirms that also small clusters exhibit a robust hysteresis and can be used for neuronal memory. Cooperativity is inherently local - only close-by channels can interact - and is therefore restricted to small assemblies of channels (Gutkin and Ermentrout, 2006). In these small clusters, spontaneous channel fluctuations become a prominent source of noise, a phenomenon occluded by the assumption of all-to-all coupling in previous studies (Naundorf et al., 2006; Zarubin et al., 2012). In contrast, the clustered model here captures channel noise and therefore predicts that the lifetime of the open and closed cluster state decreases in smaller clusters. However, we find that experimentally observed cluster sizes of about 10 channels (Moreno et al., 2016) - each gating in the millisecond range - suffice for memory on the timescale of seconds. Consequently, also small clusters offer a robust memory and, in contrast to one large cluster with only two states, the ensemble of small clusters yields a more versatile response and has a larger memory capacity.

### Ion channel cooperativity as a mechanism for graded persistent activity

Neurons can exploit these hysteretic cluster macrochannels as a short-term, analogue memory of recent voltage history - spikes open clusters and strong hyperpolarization closes them. In particular, the ensemble of clusters can differentiate stimulation strength - very strong or prolonged firing opens more clusters - so that the level of persistent firing depends on the stimulation history. In this way, the collection of cooperative clusters offers the memory capacity required for forms of graded persistent activity previously observed in neurons from entorhinal (Egorov et al., 2002), perirhinal (Navaroli et al., 2012) and prefrontal cortices (Winograd et al., 2008), as well as the amygdala (Egorov et al., 2006).

Our analysis predicts the following prerequisites for persistent activity. First, the cooperative channels should have a bistable range which comprises the resting membrane potential and whose borders set the voltage activity needed to switch the persistent activity on and off (see Figure 3 and Figure 4). In this way, as our dynamic clamp experiments in the perirhinal cortex showed, the memory is robust for multiple seconds and can be reliably controlled via neural activity. Second, for *graded* persistent activity, the channels should have a slow time constant, on the order 50–100 ms matching the maximal persistent firing frequency. Slow cooperative channels enable a gradual opening of individual clusters during stimulation, prevent progressing excitation during the persistent period and make the clusters more robust against fluctuation-driven spikes. If the time constant was too fast, all clusters would open with the voltage elevation of the first action potential and, consequently, only one persistent response state (i.e. all clusters open) would be possible.

The type of ion conducted by cooperative channels, however, is less constrained. In principle, persistent activity can be mediated by both depolarizing and hyperpolarizing cooperative channels like calcium or potassium channels, respectively (see Figure 4—figure supplement 1). If clusters consist of depolarizing calcium channels, persistent activity starts after strong spiking as observed by Egorov in the entorhinal cortex (Egorov et al., 2002). If on the other hand, clusters consist of hyperpolarizing potassium channels, persistent activity starts after strong hyperpolarization - similar to hyperpolarization-activated GPA as reported by Winograd in prefrontal cortices (Winograd et al., 2008). Independent of the ion type, we expect that the cooperative channels are separate from the action potential-generating sodium and potassium channels, or at least form only a small subgroup therein. This separation would prevent memory from interfering with the action potential, the first based on persistent currents and the latter on regenerative, memoryless currents.

### Experimental evidence and comparison to other model of persistent activity

To date, it is not known, whether neurons exhibiting graded persistent activity express cooperative ion channels. However, in mammalian cells from the hippocampus, a cooperative variant of CaV1.3, a calcium channel wide spread in the brain, has been demonstrated to provide persistent depolarizing currents and increase the firing rates of hippocampal neurons (Moreno et al., 2016). Other candidates implicated in persistent activity, the transient receptor potential cation (TRPC) channels (Zhang et al., 2011), are known to cluster and therefore provide the spatial proximity required for cooperativity (Nilius and Owsianik, 2011). However, a recent study showed that graded persistent activity in the entorhinal cortex of mice does not require TRPC channels (Egorov et al., 2019). Generally, the channels required for persistent activity are still under debate. As our work demonstrates, only a small fraction of channels has to be cooperative to produce persistent activity, so that they might be easily overlooked, especially if cooperativity depends on other intracellular regulators like calmodulin (Moreno et al., 2016).

As a model of persistent activity, cooperativity shares positive feedback as the core principle with other hypothesis like calcium modulated conductances, but differs in several aspects. The central difference is that coupled channels have an inherent, direct feedback mechanism - one channel opening facilitates another - whereas independent channels require an indirect interaction. Accordingly, previous studies suggested a feedback cycle of spiking, calcium inflow and a calcium modulated conductance as the basis of persistent spiking (Rodriguez et al., 2018). A direct consequence is that memory in the case of calcium modulated channels requires spiking, whereas cooperative clusters could also implement a silent memory - a long lasting change of excitability after stimulation. However, silent memories are also possible in extended models of calcium modulation, where a rise in calcium triggers persistent conductance changes through intracellular signaling (Fransén et al., 2006; Winograd et al., 2008). Another difference is that models based on calcium modulation naturally address the finding that blocking calcium channels or strong calcium buffering prevents persistent activity (Egorov et al., 2002; Cui and Strowbridge, 2018). For the cooperativity hypothesis, this central role of calcium could imply a calcium regulated coupling, like in the case of calcified calmodulin mediated channel interactions (Moreno et al., 2016).

As mediators of graded persistent activity, clusters of cooperative channels could be the computational substrate of important cognitive processes such as short term memory and evidence accumulation (Zylberberg and Strowbridge, 2017). Beyond graded persistent activity, the cluster bistability may serve other plasticity mechanism of intrinsic excitability such as dendritic attenuation or boosting of synaptic inputs (Marder et al., 1996; Debanne et al., 2019).

### Cooperativity dynamically regulates the computational repertoire of ion channels

An attractive feature of cooperative channels is the computational flexibility. For a cell-intrinsic memory on demand, biophysical modulators could turn on and off the cluster bistability. Bistability only emerges, when the channels are coupled strongly and the cluster consists of a sufficient number of channels. Thus, one regulatory mechanism could be to change the coupling as in the case of calcium regulated cooperativity (Moreno et al., 2016). Another regulatory mechanism could be to control clustering of channels, which is subject to factors like neural activity (Misonou et al., 2004), extracellular pH (Sumino et al., 2014), ionic concentrations (Eisenach et al., 2014) and lipid signaling (Hilgemann et al., 2018). As a composite of multiple channels, a cluster macrochannel is a more flexible conductance than its ‘hard-coded’ parts.

The emergence of memory in a cluster of cooperative, but memoryless channels suggests a general role of ion channel cooperativity. Cooperative interactions guide the formation of novel macrochannels with a gating repertoire absent at the single channel level. At the level of the neuron, the common membrane potential orchestrates sodium and potassium channel gating to generate the spike. In the same way, cooperativity could orchestrate gating in small channel assemblies to enrich neural dynamics. Correspondingly, it has been suggested that different TRP channels from heteromultimeres to create a wide variety of functions (Nilius and Owsianik, 2011). Another example of a heterogenous cluster is the assembly of BK and CaV, 1.3 channels (Berkefeld et al., 2006; Vivas et al., 2017). A quantitative understanding of these channel complexes requires detailed experimental characterization of the channel couplings like in Sato et al. (2018). Still, simple coupling models like the one presented here already reveal the potential of cooperativity to provide emergent gating functions.

In summary, clusters of cooperative channels broaden the computational repertoire of neurons. Extrapolating from the current study on cell-intrinsic memory, ion channel cooperativity can mediate direct feedback loops between channels and therefore could allow to form macrochannels with novel gating dynamics. If, additionally, neuromodulation can control cooperative interactions, ion channel cooperativity provides an extremely versatile cell-intrinsic mechanism to enrich and regulate neural activity.

## Materials and methods

### Neuron and channel model

#### Isolated ion channel model

We assume that the cooperative channels have a single activation gate and model their gating dynamics in isolation according to the calcium channel dynamics from the Morris-Lecar model (Morris and Lecar, 1981).

The activation kinetics are

(1) $$\frac{\text{d}\,m}{\text{d}\,t}=\frac{m\,\left(V\right)-m}{\tau \,\left(V\right)}$$

where the steady state activation is

(2) $$m\,\left(V\right)=\frac{1}{2}\,\left(1+\tanh\left(\frac{V-V_{1/2}}{k}\right)\right),$$

and the activation time constant reads

(3) $$\tau \,\left(V\right)=\tau \,\cosh^{-1}\left(\frac{V-V_{m}}{\sigma }\right).$$

Correspondingly, the channel has two states, open (O) and closed (C) and their kinetics reads

$$C\overset{\alpha (v)}{\underset{\beta (v)}{⇌}}O,$$

with opening rate

$$\alpha \,\left(V\right)=\frac{m\,\left(V\right)}{\tau \,\left(V\right)},$$

and closing rate

$$\beta \,\left(V\right)=\frac{1-m\,\left(V\right)}{\tau \,\left(V\right)}.$$

The channels have a single channel conductance $g_{c\,o\,o\,p}$ and reversal potential $E_{c\,o\,o\,p}$. Original parameters are $E_{c\,o\,o\,p}=100\,m\,V$, $V_{1/2}=-1\,m\,V$, k=15⁢m⁢V, τ=0.05⁢ms, $V_{m}=-1\,m\,V$ and σ=30⁢m⁢V, see Zarubin et al. (2012). Unless reported otherwise, we choose a single channel conductance of $g_{c\,o\,o\,p}=2.5\,\mathrm{pS}$ as reported for calcium channels (Church and Stanley, 1996). Modifications of these parameters are summarized in Table 1.

#### Cooperativity model

In order to capture cooperative interactions among channels, we model the activation of a channel as dependent on both the membrane potential and the state of near-by channels (Naundorf et al., 2006). Specifically, we assume that the activation $\tilde{m}$ of a channel among o open neighbours has the form

(4) $$\tilde{m}\,\left(V,o\right)=m\,\left(V+o\,j\right).$$

where j is the *coupling strength between two channels*. Therefore, if the coupling is positive j>0, opening of a neighbouring channel shifts the activation curve towards lower membrane potentials. As a result, a channel with open neighbours has itself an increased open probability, see Figure 1A.

For the time constant, we posit that the same shift applies,

(5) $$\tilde{\tau }\,\left(V,o\right)=\tau \,\left(V+o\,j\right).$$

#### Cluster model

Next, we introduce the experimentally observed clustering of the channels. We presume that only channels in the same cluster are close enough to cooperate, so that channels in different clusters gate independently. For a cluster, a characteristic measure of cooperativity is the *maximal shift *J, which corresponds to the shift of the activation curve when all neighbours of a channel are open. Under the simplifying assumption of constant coupling strength j among all channels, the maximal shift in a cluster of size S amounts to J=(S-1)⁢j.

A note on comparing coupling strengths with previous studies: We choose J for the maximal shift in accordance with the notation of Zarubin et al. (2012). In contrast, Naundorf et al used J to denote the coupling strength between two channels, which in the work presented here is j (Naundorf et al., 2006).

In a cluster of size S with o open channels, all S-o closed channels open with rate

$$\tilde{\alpha }\,\left(V,o\right)=\alpha \,\left(V+o\,j\right)$$

and all o open channels, having only o-1 open neighbours, close with rate

$$\tilde{\beta }\,\left(V,o-1\right)=\beta \,\left(V+\left(o-1\right)\,j\right).$$

Instead of tracking the cluster state in terms of each constituting channel, we can also view the cluster as a macrochannel. For S two-state channels, this macrochannel has S+1 conductance states, from all channels closed to all channels open. In a small time interval, at most one channel opens or closes, so that transitions are restricted to adjacent cluster states with a difference of one open channel,

$$o\overset{\alpha_{o,o+1}(v)}{\underset{\beta_{o+1,o}(v)}{⇌}}o+1.$$

The macrochannel rates result from the number of channels that are possible candidates for the transition and the single channel transition rate. Therefore, in a cluster with o open channels, the opening of one of the other S-o closed channels happens with rate

(6) $$\alpha_{o,o+1}\,\left(V\right)=\left(S-o\right)\,\alpha \,\left(V+o\,j\right).$$

Correspondingly, from a state with o+1 open channels, the closing of one of the open channels each having o open neighbours reads

(7) $$\beta_{o+1,o}\,\left(V\right)=\left(o+1\right)\,\beta \,\left(V+o\,j\right).$$

Figure 1C shows simulated traces of clusters and demonstrates their behaviour as macrochannels.

#### Neuron model

We used a conductance-based neuron model with a single isopotential compartment,

(8) $$C\,\frac{d\,V}{d\,t}=I_{\mathrm{app}}-I_{\mathrm{cluster}}-I_{V},$$

where $I_{\mathrm{cluster}}$ is the current through the clusters of cooperative channels, $I_{V}$ summarizes the other channel currents, $I_{\mathrm{app}}$ is a stimulus current and C is the capacitance of the membrane.

### Cluster current

We model the cooperative channels arranged in N clusters, each composed of S identical channels. Then, the current through all clusters is determined by the total number of open channels $O_{\mathrm{coop}}$ among all clusters,

(9) $$I_{c\,l\,u\,s\,t\,e\,r}=g_{c\,o\,o\,p}\,O_{\mathrm{coop}}\,\left(V-E_{c\,o\,o\,p}\right),$$

where $E_{c\,o\,o\,p}$ denotes the reversal potential of the considered ions. For most of the article, we assume that the cooperative channels conduct a depolarising current with $E_{c\,o\,o\,p}=100\,m\,V$ (e.g. Ca2+ or Na+). In Figure 4—figure supplement 1, we then consider the case of a hyperpolarising current with $E_{c\,o\,o\,p}=-100\,m\,V$ (e.g. K+).

In a description, where each channels is tracked, $O_{\mathrm{coop}}$ simply counts the number of channels in the open state. In the alternative macrochannel description of the clusters, the total number of open channels is obtained from the number of macrochannels in the different conductance states, so

$$O_{\mathrm{coop}}=\sum_{o=0}^{S}\gamma_{o}\,o,$$

where $\gamma_{o}$ is the number of clusters with o open channels. This occupancy vector γ can also be used to capture the distribution of conductance states of a single cluster over time (Figure 1C).

As opposed to the other ionic conductances, the dynamics of the clusters is modeled on the level of the underlying jump process generated by the single-channel gating events. Such a detailed description is necessary to account for the fact that cooperative interactions reduce the number of independent stochastic units and therefore increase fluctuations ($1/\sqrt{N}$ as opposed to $1/\sqrt{S\,N}$ White et al., 2000). Effectively, the N clusters represent the independent units, so that with around 100 clusters, a jump process description is adequate to account for the discrete nature of the fluctuations.

### Leak and action potential mediating currents

We choose a type 1 neuron model to account for the continuous low-frequency firing range as observed in graded persistent activity (Egorov et al., 2002). The model presented is a version of the Traub-Miles model (Benda, 2002), which comprises action potential generating potassium and sodium currents and a leak current,

$$I_{V}=A\,\left(I_{N\,a}+I_{K}+I_{L}\right).$$

The original model is formulated with conductance densities and is independent of the neuron surface area A. In the present model, the cluster conductance is defined in absolute terms, because it stems from a concrete number of channels with a fixed conductance. Correspondingly, we turn the remaining currents and the capacitance into absolute quantities by choosing a surface area, for details see Choosing a neuron surface. The capacitance density is $\bar{C}=1\,\mu \,F/{\mathrm{cm}}^{2}$.

In the following, all gating variables have first order kinetics of the form

(10) $$\dot{x}=\alpha_{x}\,\left(V\right)\,\left(1-x\right)-\beta_{x}\,\left(V\right)\,x.$$

#### Sodium current

$$I_{N\,a}={\bar{g}}_{N\,a}\,m^{3}\,h\,\left(V-E_{N\,a}\right)$$

with ${\bar{g}}_{N\,a}=100\,\frac{\mathrm{mS}}{{\mathrm{cm}}^{2}}$, $E_{N\,a}=48\,m\,V$ and gating variables m and h with rates

$$\alpha_{m}\,\left(V\right)=0.32\,\mathrm{kHz}\,\left(V/\mathrm{mV}+54\right)/\left(1-\exp\left(-0.25\,\left(V/\mathrm{mV}+54\right)\right)\right),$$

$$\beta_{m}\,\left(V\right)=0.28\,\mathrm{kHz}\,\left(V/\mathrm{mV}+27\right)/\left(\exp\left(0.2\,\left(V/\mathrm{mV}+27\right)\right)-1\right),$$

$$\alpha_{h}\,\left(V\right)=0.128\,\mathrm{kHz}\,\exp\left(-\left(V/\mathrm{mV}+50\right)/18\right),$$

$$\beta_{h}\,\left(V\right)=4.0\,\mathrm{kHz}/\left(\exp\left(-0.2\,\left(V/\mathrm{mV}+27\right)\right)+1\right).$$

#### Potassium current

$$I_{K}={\bar{g}}_{K}\,n^{4}\,\left(V-E_{K}\right)$$

with ${\bar{g}}_{K}=200\,\frac{\mathrm{mS}}{{\mathrm{cm}}^{2}}$, $E_{K}=-82\,m\,V$ and gating variable n with rates

$$\alpha_{n}\,\left(V\right)=0.032\,\mathrm{kHz}\,\left(V/\mathrm{mV}+52\right)/\left(1-\exp\left(-0.2\,\left(V/\mathrm{mV}+52\right)\right)\right),$$

$$\beta_{n}\,\left(V\right)=0.5\,\mathrm{kHz}\,\exp\left(-\left(V/\mathrm{mV}+57\right)/40\right).$$

#### Leak current

$$I_{L}={\bar{g}}_{L}\,\left(V-E_{L}\right)$$

with ${\bar{g}}_{L}=0.1\,\frac{\mathrm{mS}}{{\mathrm{cm}}^{2}}$ and $E_{L}=-67\,m\,V$.

#### Choosing a neuron surface

When we fix the peak conductances of the other ionic currents, the contribution of the cluster current for a fixed number of cooperative channels depend on the neuron area A. For example, for a small neuron area, the cluster conductance becomes relatively larger and could drive faster persistent spiking than for a large neuron area. In choosing a neuron area, we try to meet two characteristics of graded persistent activity, namely its low-frequency range and its quasi-continuous nature.

Specifically, the frequency of persistent firing is usually located in the low-frequency range below 15 Hz, which sets a limit to the conductance of the clusters. In terms of conductance densities, we find that close to rheobase $I_{a\,p\,p}=0.105\,\mu \,A/{\mathrm{cm}}^{2}$ and with a reversal potential $E_{c\,o\,o\,p}=100\,m\,V$ common for Ca_2+_ channels, a cooperative channel conductance density of about ${\bar{g}}_{\mathrm{coop}}\approx 0.0004\,\frac{\mathrm{mS}}{{\mathrm{cm}}^{2}}$ is sufficient to drive spiking at about 10 Hz.

Furthermore, the levels of persistent firing are assumed to be quasi-continuous, which requires a large number of clusters with a small conductance. In all simulations, we assumed about 100 clusters, which in principle allow 100 levels of persistent firing. In a frequency range of up to 10 Hz, this would correspond roughly to a frequency resolution of 0.1 Hz, which lies below the experimentally observed grading of about 0.5 Hz (Fransén et al., 2006). Furthermore, the clusters have to be of a certain size to be bistable on the timescales of seconds, as shown in Figure 3. Hence, for a high number of clusters N = 100 with size S = 8 and a typical single channel conductance g=2.5⁢pS, the total cooperative channel conductance is $g_{\mathrm{coop}}=2\,\mu \,S$.

Therefore, we arrive at a required surface area of,

$$A=\frac{g_{\mathrm{coop}}}{{\bar{g}}_{\mathrm{coop}}}=0.005\,{\mathrm{cm}}^{2},$$

which is a large neuron area compared to typical neuron surface areas as reported in literature (e.g. Ambros-Ingerson and Holmes, 2005). This large membrane area also explains the rather high currents that are needed to excite and hyperpolarise the neuron. In principle, a smaller neuron area could be reached by smaller cluster numbers, smaller cluster sizes, a lower reversal potential or an increase of the firing range.

#### Stimulation with a white noise current

We investigate how robust the clusters are against noise in the membrane potential (Schreiber et al., 2009). In particular, we mimic fluctuating synaptic input to the neuron, a major noise source, by injecting a white noise current with standard deviation $\sigma_{I}$ and time resolution ${\text{dt}}_{n\,o\,i\,s\,e}$,

$$I_{n\,o\,i\,s\,e}\,\left(t\right)=\sum_{k}i_{k}\,\left(H\,\left(t-k\,{\text{dt}}_{n\,o\,i\,s\,e}\right)-H\,\left(t-\left(k+1\right)\,{\text{dt}}_{n\,o\,i\,s\,e}\right)\right);i_{k}\,\text{from }\,𝒩\,\left(0,\sigma_{I}^{2}\right),$$

where H⁢(x) is the Heaviside step function and $𝒩\,\left(0,\sigma_{I}^{2}\right)$ denotes a normal distribution with zero mean and variance $\sigma_{I}^{2}$.

We choose a time resolution of ${\text{dt}}_{n\,o\,i\,s\,e}=0.5\,m\,s$, oriented at the temporal width of synaptic currents, and then vary the noise intensity (see Figure 4—figure supplement 2). Note that the simulation step size is much smaller than the temporal resolution of the noise (see Simulation of the neuron model).

Overview of cluster and cooperative channel parameters.

### Simulation and analysis of cluster dynamics

#### Mean channel activation in a cluster

In a large cluster of cooperative channels, the number of open channels is expected to coincide with its mean $\bar{o}=m_{\mathrm{coop}}\,\left(V\right)\,S$, where $m_{\mathrm{coop}}\,\left(V\right)$ is the average activation of the channels in the cooperative ensemble. Moreover, the average activation reflects how much the open neighbours shift the activation curve,

$$m_{\mathrm{coop}}\,\left(V\right)=m\,\left(V+\bar{o}\,j\right),$$

where we use Equation 4 for the cooperativity altered activation and m⁢(V) is the single channel activation function. A rewrite of the shift term $\bar{o}\,j=m_{\mathrm{coop}}\,\left(V\right)\,S\,j\approx m_{\mathrm{coop}}\,\left(V\right)\,J$ results in a self-consistency relation (Naundorf et al., 2006, Supplementary Notes 2),

(11) $$m_{\mathrm{coop}}\,\left(V\right)=m\,\left(V+m_{\mathrm{coop}}\,\left(V\right)\,J\right).$$

We numerically solve Equation 11 for a range of membrane potentials to obtain the activation curves $m_{\mathrm{coop}}\,\left(V\right)$ for different couplings J. As discussed in the results on cluster dynamics, the activation becomes bistable for sufficiently strong coupling $J>J_{c\,r\,i\,t}$ (Figure 1).

The critical coupling strength depends on the form of the single channel activation m⁢(V). For the activation curve in Equation 4, it reads (derivation below)

$$J_{c\,r\,i\,t}=2\,k.$$

Hence, the critical coupling $J_{c\,r\,i\,t}$ coincides with the width of the activation curve. Put differently, bistability emerges, when cooperative facilitation by the neighbouring open channels can keep a channel open despite a low membrane potential at which the channel would usually be closed. Thus, when all neighbours are open, the shift has to exceed the width of a channel’s activation curve (Figure 1A).

### Derivation of the critical coupling strength

For the derivation of the critical coupling strength, we follow the argument of Huang et al. (2012), (Equations 4-10). They consider cooperative sodium channels and are interested in the coupling strength, where the channel activation becomes a step function. This coupling strength in principle also induces bistability; the steps in the activation curve occur at the edges of the bistable range. However, sodium channels transit to the inactivated state when they are open and thus they cannot be bistable.

If a bistable range exists, the number of solutions of the self-consistency Equation 11 has to change from one to three (bistable) and back to one again, when the voltage is increased. From this observation, we can deduce a condition in the coupling strength J. Namely, each solution $m_{c}$ corresponds to an intersection of the left and right hand of Equation 11,

$$m_{c}=m\,\left(V+m_{c}\,J\right)$$

and for the voltages where the number of solutions change, these intersections have to be tangential, that is

$$\frac{d}{dm_{c}}m_{c}=\frac{d}{dm_{c}}m(V+m_{c}J)$$

$$\Rightarrow \;1=\frac{J}{2k}\left(1-{\left(2m(V+m_{c}J)-1\right)}^{2}\right).$$

Here, we use that $\frac{\text{d}\,m\,\left(V\right)}{\text{d}\,V}=\frac{1}{2\,k}\,\left(1-{\left(2\,m\,\left(V\right)-1\right)}^{2}\right)$. Again using the self-consistency relation $m_{c}=m\,\left(V+m_{c}\,J\right)$, we observe that the solution has to obey

$$\frac{J}{k}\,m_{c}^{2}-\frac{J}{k}\,m_{c}+\frac{1}{2}=0.$$

Finally, as the solution has to be real, bistability sets the following condition on $J:{\left(\frac{J}{k}\right)}^{2}-2\frac{J}{K}\geq 0\Rightarrow J\geq 2k=J_{crit}.$

### Mean first passage times between cluster states

In order to study the stability of the open and closed cluster state, we calculate the mean first passage times between these two states. We employ the macrochannel description, where for a cluster of size S the dynamics form a continuous-time Markov process with transition matrix

$$Q_{i\,j}\,\left(V\right)=\delta_{i,j+1}\,\alpha_{i-1,i}\,\left(V\right)+\delta_{i,j-1}\,\beta_{i+1,i}\,\left(V\right);0\leq i,j\leq S.$$

$Q_{i\,j}\,\left(V\right)$ denotes the voltage-dependent transition rate from cluster state j to i and summarizes the opening and closing rates from Equation 6 and 7.

Over a small time interval, the transition rates become transition probabilities and the continuous Markov chain can be discretized. That is, choosing a small time interval Δ⁢t, such that for all i,j it holds $Q_{i\,j}\,\Delta \,t\ll 1$, the transition probabilities are $P_{i\,j}=Q_{i\,j}\,\Delta \,t$ for i≠j and $P_{i\,i}=1-\sum_{j\neq i}P_{j\,i}$. For a discrete Markov chain, the mean first passage steps K (with $K_{i\,j}$ denoting the mean number of steps required to reach state i from state j) ) can be obtained by solving the system of equations given by Allen (2010), see p.69)

K=E+(K-d⁢i⁢a⁢g⁢(K))⁢P,

where E is a (S+1)×(S+1) matrix of ones. Multiplication with the time interval recovers the mean first passage times M=K⁢Δ⁢t.

For the stability of a bistable cluster, the mean first passage times between the open and closed state are of particular interest: the average time ${\bar{\tau }}_{O\to C}$ it takes until a cluster spontaneously switches from the state with all channels open to the one with all channels closed or vice versa ${\bar{\tau }}_{C\to O}$. In terms of M, they read ${\bar{\tau }}_{O\to C}=M_{0,S}$ and ${\bar{\tau }}_{C\to O}=M_{S,0}$. Figure 3A depicts the mean residence times as a function of the membrane voltage.

#### Numerical simulation of cluster dynamics

For the simulation of the cluster dynamics at a constant membrane potential, we use both a fixed time step method and the Gillespie algorithm (Gillespie, 1977).

In the fixed time step method, we track the state of each channel in the cluster. For each time step, we look up the number of open channels, evaluate the corresponding opening and closing rates and obtain the transition probabilites by multiplying the rates with the time step. Finally, a random number generator is used to update the state of the channels according to the transition probabilities. This method is used for the cluster simulations in Figure 1 and Figure 2.

For the application of the Gillespie algorithm (Gillespie, 1977), we switch to the macrochannel description, where the states only specify the number of open channels, but ignore the configuration of the individual channels. Specifically, for clusters of size S, the S+1 conductance states represent the species in the Gillespie algorithm, whereas the 2⁢S transitions between adjacent cluster states with their respective rates form the reactions.

### Simulation of the neuron model

In the neuron model, the coupled dynamics of the continuous variables, voltage (Equation 8) and gating variables (Equation 10), and the jump process of the cooperative channels form a hybrid stochastic system (Anderson et al., 2015). For the simulation of spiking activity, we use brute, a custom written fixed time step algorithm.

#### BRUTE algorithm

The brute algorithm tracks the state of each channel and uses a fixed time method to update their states. In order to take into account the cooperative interactions among channels in one cluster, it considers each cluster separately and calculates the opening and closing rate of the channels therein. For N clusters of size S, the channel states can be cast into a matrix $C\in {\left\{0,1\right\}}^{N\times S}$, which the algorithm updates in each time step.

In the following, the membrane potential and the continuous gating variables are summarized in 𝐱 and the current time is t. Channel states are encoded as 0 (closed) or 1 (open). Then, in pseudo code, the update algorithm with time step Δ⁢t is

1. Initialize: set $t=t_{0}$, set the initial membrane potential and gating variables ${𝐱}_{0}$ and set the initial channel states $C\in {\left\{0,1\right\}}^{N\times S}$
2. Generate an array of random numbers $r\in {\left[0,1\right]}^{N\times S}$
3. Numerically integrate voltage and gating variables dynamics (Equation 8; 10) until t+Δ⁢t
4. Update each cluster, that is each row i of C as follows
  1. calculate the number of open channels $o_{i}=\sum_{j}C_{i\,j}$
  2. update each channel $C_{i\,j}$
    - if $C_{i\,j}=1$ and $r_{i\,j}<\beta \,\left(V,o_{i}-1\right)\,\Delta \,t$ then $C_{i\,j}=0$
    - else if $C_{i\,j}=0$ and $r_{i\,j}<\alpha \,\left(V,o\right)\,\Delta \,t$ then $C_{i\,j}=1$
5. Increment t by Δ⁢t and check if simulation is finished, otherwise continue with step 2

In all simulations, we chose a time step Δ⁢t=0.25⁢u⁢s. At this small time resolution, we observed a convergence of the cluster dynamics. A python implementation of the algorithm with a simple example is available at https://itbgit.biologie.hu-berlin.de/cooperativity/brutelib.git (copy archived at https://github.com/elifesciences-publications/49974-brutelib).

### Dynamic clamp experiment

#### Preparations of mouse brain slices

Dynamic clamp experiments were conducted with horizontal mouse brain slices (300–450 µm thick) containing the hippocampus, entorhinal and perirhinal cortices. Brain slices were obtained from male C57BL/6N mice (4–8 weeks old, 20–25 g) using standard proceedings (Roth et al., 2016). Mice were purchased from Charles River Laboratories (Sulzfeld, Germany, Strain Code: 027) and were taken care of in the Interfaculty Biomedical Research Facility in Heidelberg. Housing was provided in Makrolon II cages with a maximum of three animals and tissue nesting material made of cellulose. Animals had ad libitum access to food and water. All experimental protocols were conducted in compliance with German law and with the approval of the state government of Baden-Württemberg (Project T100/15).

In order to minimize the stress of euthanasia, mice were sedated by exposure to CO_2_ in a rising concentration (20–30 l/h sourced from a compressed gas cylinder) until the animal fell unconscious. Mice were subsequently killed by decapitation and the brain was quickly removed and transferred to 4°C cold, carbogen buffered (95% O_2_, 5% CO_2_ at pH 7.4) artificial cerebrospinal fluid (ACSF) containing the following (in mM): 124 NaCl, 3 KCl, 1.8 MgSO_4_, 1.6 CaCl_2_, 10 glucose, 1.25 NaH_2_PO_4_, 26 NaH_2_CO_3_. Brain slices were cut using a vibratome (Leica VT1200S, Nussloch, Germany). Then, slices were transferred to a Haas-type interface chamber (Haas et al., 1979), perfused with ACSF at a rate of 1.2–1.4 ml/min at 34 ±1°C. Slices rested for at least 2 hr before electrophysiological recordings.

#### Electrophysiological recordings

Single-cell recordings were obtained from principal neurons of the perirhinal cortex layer II. These neurons show graded persistent firing under activation of muscarinic cholinergic receptors (Navaroli et al., 2012).

The recordings were obtained with sharp microelectrodes (tip resistance 100–130 MΩ) pulled from 1.0 mm borosilicate glass capillaries (Harvard Apparatus, Cambridge, UK, Cat. No. 30–0019) on a DMZ Universal Electrode Puller (Zeitz, Martinsried, Germany) and filled with 2 M K-acetate that in some cases contained 1% biocytin.

Recorded signals were low-pass filtered at 10 kHz, amplified x10 using an SEC-05X amplifier (npi electronic, Tamm, Germany) and digitized at 20 kHz with an analog to digital converter (MICRO 1401 mkII ADC, CED, Cambridge, UK). Signals were visualized and saved using Spike2 software (CED, Cambridge, UK). All intracellular recordings were conducted in bridge mode and bridge balance was monitored and adjusted during experiments.

Positive and negative current pulses (duration and amplitudes controlled by Spike2 Software and SEC-05X amplifier) were applied via the recording electrode to determine input resistance and firing properties. Input resistance was estimated during the experiment and later calculated off-line using Matlab software (The Mathworks, Natick, MA). Resting membrane potential was calculated by subtracting the potential offset after withdrawal from the cell at the end of the recording.

All cells selected for measurements had an input resistance above 20 MΩ, a stable membrane potential throughout the recording and exhibited firing properties of principal neurons. Membrane potential of neurons was controlled by manually adjustable DC current injection through the recording electrode to determine the current needed for intended firing frequencies and depolarisation near threshold.

To prevent spontaneous network activity in slices, all experiments were performed in the presence of ionotropic glutamate receptor blockers 6-cyano-7-nitroquinoxaline-2,3-dione (CNQX, 30 µM, Cat. No. 1045) and DL-2-amino-5-phosphonovaleric acid (APV, 10 µM, Cat. No. 0105) obtained from Tocris (Bristol, UK). Drugs were bath applied by continuous perfusion. Biocytin (Cat. No. B4261) was obtained from Sigma-Aldrich (Taufkirchen, Germany).

### Biocytin staining

After recordings the slice was fixed in 4% PFA in phosphate buffer (PB) at 4°C for 90 min and then stored in phosphate buffered saline (PBS) at 4°C until further processing. It was washed 3 × 15 min to remove excess PFA and incubated with Avidin - Alexa Fluor 488 conjugate (1:1000; Life Technologies, Carlsbad, CA) diluted in PBS containing 5% normal goat serum and 0.2% Triton X at room temperature under light protection for 2 hr. The slice was then washed in PBS for 15 min, incubated with 4,6-diamidino-2-phenylindole (DAPI; 1:10,000; Carl Roth, Germany) in H_2_O for 3 min, washed again in PBS (15 min) all at room temperature and afterwards embedded in Mowiol 4–88 (Sigma-Aldrich, Taufkichen, Germany).

Fluorescence images were acquired with a Nikon A1+ Confocal Microscope (Nikon, Düsseldorf, Germany) and reconstruction of the neuron was obtained from z-stack confocal images using Neurolucida tracing software (MBF Bioscience, Williston, VT).

#### Dynamic clamp

Data acquisition and dynamic clamp loop were controlled by RELACS, V0.9.8, RRID:SCR_017280. The feedback loop run at a frequency of 20 kHz and consisted of sampling the membrane potential, updating the state of the clusters of cooperative channels and injection of the corresponding current (Equation 9). The software allowed online adjustments of the following model parameters:

- the channel to channel coupling strength j
- cluster number N and cluster size S
- single channel conductance $g_{c\,o\,o\,p}$ and reversal potential $E_{c\,o\,o\,p}$
- parameters of the channel kinetics, namely maximum of the time constant τ and the half-activation voltage $V_{1/2}$

### Real time cluster update in the dynamic clamp loop

In order to meet the real time requirement imposed by dynamic clamp, we employ the macrochannel description of the clusters (see Cluster model). In each time step, the macrochannel description updates the cluster population in the S+1 conductance states of a cluster by evaluating the 2⁢S possible transitions. This provides a huge reduction of transitions, when compared to the N⋅S state update required to each channel.

In pseudo code, the update algorithm works as follows:

1. Initialize: set initial cluster population $\gamma_{0}\in {\left\{0,\ldots ,N\right\}}^{S+1}$
2. Get currently measured membrane potential V
3. Generate two arrays of random numbers $r^{o\,p\,e\,n}\in {\left[0,1\right]}^{S}$ and $r^{c\,l\,o\,s\,e}\in {\left[0,1\right]}^{S}$ for the 2⁢S opening and closing reactions
4. Calculate propensities of the 2⁢S reactions via the macrochannel transition rates in Equation 6 and Equation 7.
  - $\lambda_{o}^{o\,p\,e\,n}=\gamma_{o}\,\alpha_{o,o+1}\,\left(V\right)$ for o∈{0,S-1}
  - $\lambda_{o}^{c\,l\,o\,s\,e}=\gamma_{o+1}\,\beta_{o+1,o}\,\left(V\right)$ for o∈{0,S-1}
5. Update cluster populations
  - S opening reactions: if $r_{o}^{o\,p\,e\,n}<\lambda_{o}^{o\,p\,e\,n}\,\Delta \,t$ then increase $\gamma_{o+1}$ by one and decrease $\gamma_{o}$ by 1
  - S closing reactions: if $r_{o}^{c\,l\,o\,s\,e}<\lambda_{o}^{c\,l\,o\,s\,e}\,\Delta \,t$ then increase $\gamma_{o}$ by one and decrease $\gamma_{o+1}$ by 1
6. Wait until the time interval Δ⁢t has passed and continue at step 2

The successive update of the cluster populations can lead to a problem for states, which have a population of one and where both the opening and the closing reaction happen. In our solution, we chose to assure a positive population for all states and reject the closing reaction in such a case. In an offline comparison with the exact Gillespie algorithm, we tested that our update algorithm accurately captured the evolution of the clusters despite this slight bias toward opening reactions.

The algorithm with instructions how to use it in the recording software RELACS is available at https://itbgit.biologie.hu-berlin.de/cooperativity/dynamic_clamp_model (copy archived at https://github.com/elifesciences-publications/49974-dynamic_clamp_model).

#### Experimental protocol

In all recordings, we first measured the f-I curve of the neuron and determined the resting potential and the form of the action potentials. With these neuron properties at hand, we chose the model parameters for the dynamic clamp experiment. First, we aimed at a total conductance of the cooperative channels, that allowed a current to flow sufficient for about 10 Hz persistent firing. Correspondingly, we chose the number of cooperative channels, given that channel conductance and reversal potential were orientated at values from calcium channels. Second, we split the channels into clusters such that we obtained both a large number of clusters and a sufficient cluster size for long-term stability. Third, we adjusted the bistable regime of the clusters such that its center coincided with the resting potential and its borders remain far below the spike peak. To this end, we selected the appropriate coupling strength and half-activation voltage of the channels.

We used the stimulation protocol of consecutive pulses, either depolarising or hyperpolarising, to test for graded persistent activity. Pulses had a length of 4–6 s with a period of about 25 s. Often, we saw channels opening persistently during the depolarising pulses, but no persistent activity. In these cases, we would increase the baseline current to bring the neurons closer to threshold. Additionally, we run control protocols with zero coupling to test whether the cell displayed no persistent activity in the absence of cooperativity (Figure 6—figure supplement 1).

#### Analysis

First, we categorised the recordings according to the amplitude and the number of pulses, for example UP-4 for a series of four depolarising pulses or DOWN-1 for one hyperpolarising pulse. Then, the inter spike intervals were analysed during and after the pulses as well as before and after the pulse protocol. For the inter spike intervals, we excluded the first 1000 ms of each interval to account for transient currents. Next, we defined a simple success criteria for a protocol: during an UP (DOWN) protocol, the frequency of persistent firing increases (decreases) with each pulse. During increase or decrease successive periods of silence were allowed to include runs where the persistent firing would only start after for example the second pulse. From the three recorded cells, we got successful runs of every protocol: DOWN-1 (two successful/3 total), DOWN-3 (2/2), DOWN-4 (6/8). UP-1 (7/15), UP-3 (1/1), UP-4 (4/7). An overview of all protocols is available at https://gin.g-node.org/ppfeiffer/cooperative_channels_in_biological_neurons_via_dynamic_clamp, together with the raw data and python scripts for analysis.

For the presented recordings, the model parameters are summarized in Table 1.

## Data Availability

The model for the cooperative ion channels and the neuron are described in the manuscript. Software for simulation of the cooperative channels is provided in git repositories linked in the manuscript (https://itbgit.biologie.hu-berlin.de/cooperativity/brutelib; copy archived athttps://github.com/elifesciences-publications/49974-brutelib; and https://itbgit.biologie.hu-berlin.de/cooperativity/dynamic_clamp_model; copy archived at https://github.com/elifesciences-publications/49974-dynamic_clamp_model). The recordings and analysis obtained in the dynamic clamp experiments are explained in the manuscript and are publicly available at https://gin.g-node.org/doi/cooperative_channels_in_biological_neurons_via_dynamic_clamp. The following dataset was generated: PfeifferPEgorovALorenzFSchleimerJDraguhnASchreiberS2019Clusters of cooperative ion channels enable a membrane potential-based mechanism for short-term memoryG-Node10.12751/g-node.08853ePMC700721832031523

## Additional files

Transparent reporting form
