# Supplementary material for: Clusters of cooperative ion channels enable a membrane-potential-based mechanism for short-term memory
Source: eLife. 2020 Feb 7;9:e49974. doi: 10.7554/eLife.49974 (PMC7007218; doi:10.7554/eLife.49974)
Supplement: Peer review history

# Decision letter

**DOI:** 10.7554/eLife.49974.sa1

**Affiliations:** National Heart, Lung and Blood Institute, National Institutes of HealthUnited States; University of ConnecticutUnited States

In the interests of transparency, eLife publishes the most substantive revision requests and the accompanying author responses.

**Acceptance summary:**

In this interesting paper, the authors demonstrate via a mathematical modeling approach that cooperative gating with ion channels such as Ca^2+^ channels can be tuned to form bistable conductances that act as a potential form of memory by integrating inputs over longer periods that would be anticipated in single channels gating independently. The authors demonstrate that the integrative memory – hysteresis – allows for persistent graded neuronal firing.

**Decision letter after peer review:**

Thank you for submitting your article "Clusters of cooperative ion channels enable a membrane potential-based mechanism for short-term memory" for consideration by *eLife*. Your article has been reviewed by three peer reviewers, and the evaluation has been overseen by José D. Faraldo-Gómez as Reviewing Editor and Gary Westbrook as the Senior Editor. The following individuals involved in the review of your submission have agreed to reveal their identity: Colleen Clancy (Reviewer #2)

Although it is customary for *eLife* to condense reviewers' reports into a concise decision letter, in this case the Reviewing Editor believes it would be best to enclose these reports as originally submitted. Based on these reports, we would like to invite you to submit a revised version of your manuscript that addresses the questions and concerns raised.

Reviewer #1:

Pfeiffer et al. study the computational consequences of the cooperative on- and off-dynamics within the population of voltage-dependent ion channels. While the classic conductance-based neuron models assume that channels open and close independently, experimental evidence shows more and more cooperative effects. For example, Kim et al., 2014) has shown that potassium channel cooperativity can occur. In the present manuscript Pfeiffer et al. show a hysteresis and bistability in the gating properties of such groups of channels.

Similar to coupled magnets, a cluster of interacting ion-channels can lead to a hysteresis and a bistable opening dynamics within that cluster. On the functional level, the ion channel bistability can be exploited to implement persistent spiking in neurons that is initiated by depolarising pulse and stopped by a hypopolaring signal.

What I like about the paper is that it combines mathematical modeling with dynamic clamp experiments. Also the authors link the presence of cooperative clusters to persistent activity, something that operates at the spiking level. Somewhat counter intuitively, the cooperative coupling need not be present in the channels that are involved in the action potential generation (e.g. Na and K) but it seems that it is sufficient that cooperativity of some depolarising cationic channels in the membrane is present.

1) Can the authors comment on whether the cooperativity of depolaring channels that are involved in the AP generation leads to the same or different net-effect (bistability) as that of other depolarising cationic channels. Is there any functional difference?

2) How does the bistable switch operate in the presence of noise, are there any spontaneous switches?

How does the duration of the upper state relate to the noise level?

3) From spiking networks we know that presence or absence of bistability can depend on the size of the network. The depolaring pulse to induce a switch needs to be stronger and longer for larger networks, ~to sqrt(N). Does the size of the cluster and the size of the switch inducing pulse correlate in some way? Can the authors comment on what a realistic regime could be?

4) I think it would be a good idea to streamline the Introduction and Discussion and cut the number of figures and panels. It does seem like the last 3 figures have somewhat overlapping messages.

Reviewer #2:

In this interesting paper, the authors demonstrate via a mathematical modeling approach that cooperative gating with ion channels such as Ca^2+^ channels can be tuned to form bistable conductances that act as a potential form of memory by integrating inputs over longer periods that would be anticipated in single channels gating independently. The authors demonstrate that the integrative memory – hysteresis – allows for persistent graded neuronal firing.

The idea of coupling between ion channels as a form of memory is an elegant concept. I do wish the authors would take it a bit further: there are a number of channels that have demonstrated cooperative gating and more generally coupled gating. These vary widely from K currents to gap junctions with a variety of associated kinetics – fast versus slow gating, low versus high conductance, long versus short open times and latencies to opening etc. I think the authors could do a more expansive exploration of the potential memory dynamics that could be coded within such a large parameter space. Such an analysis may open more doors for future computational and experimental exploration.

Reviewer #3:

The authors propose a new intriguing mechanism that keeps short-term 'memory ' of recent (seconds) history of membrane potential changes by changing conductance states of clusters of cooperative channels. Changes of conductance states of cooperative channels, in turn, produce changes of the rate of persistent firing. The authors explore this novel mechanism in computer simulations and demonstrate that it can indeed mediate graded changes of persistent firing in dynamic clamp experiments with neurons from entorhinal cortex in slices.

In my view, the following aspects make this study especially important and of broad interest:

First, demonstration that cooperativity in a cluster of channels introduces a fundamentally new dynamics to the gating, expanding dramatically (by the orders of magnitude) the time scale on which channel clusters could keep trace of prior changes of the membrane potential.

Second, demonstration that such mechanism could mediate gradual changes of persistent activity of neurons – a feature that has important functional implications.

The proposed mechanism can help to establish an important link from basic electrophysiological properties of neurons and individual channels to cognitive mechanisms of short-term memory.

I have however several concerns that should be addressed.

1) What turns off open channels in cooperative clusters? Say in Figure 1, at V=V_0.5_, activation curve for strongly cooperative channels (Figure 1B) is at 1. Why would all channels close at about same time? Is their closing (inactivation?) also cooperative?

A more comprehensive analysis of opening/closing dynamics of a cluster may help here.

It is clear that a strong hyperpolarization can turn channels off, but how would this happen spontaneously?

2) Conductance through permanently open channel clusters might change the membrane potential (e.g. in Figures 4 or 5 simulations). Certainly, resulting Vm shift would depend on the ratio of conductance through channel clusters to other conductances, but since it influences firing, it might also influence the Vm.

3) Writing is often in very general terms, lacking specific details; e.g.:

– Authors talk about "channels" – which channels? If specifics of ionic conductance does not matter, this should be clearly stated in the very beginning.

– Introduction (last paragraph), it is not clear that you are talking about model simulations.

– Materials and methods: description of electrophysiological experiments is very vague. Recording from which brain area? Which cells were targeted?

# Reply: Author response

**DOI:** 10.7554/eLife.49974.sa2

Reviewer #1:

Pfeiffer et al. study the computational consequences of the cooperative on- and off-dynamics within the population of voltage-dependent ion channels. While the classic conductance-based neuron models assume that channels open and close independently, experimental evidence shows more and more cooperative effects. For example, Kim et al., 2014) has shown that potassium channel cooperativity can occur. In the present manuscript Pfeiffer et al. show a hysteresis and bistability in the gating properties of such groups of channels.

Similar to coupled magnets, a cluster of interacting ion-channels can lead to a hysteresis and a bistable opening dynamics within that cluster. On the functional level, the ion channel bistability can be exploited to implement persistent spiking in neurons that is initiated by depolarising pulse and stopped by a hypopolaring signal.

What I like about the paper is that it combines mathematical modeling with dynamic clamp experiments. Also the authors link the presence of cooperative clusters to persistent activity, something that operates at the spiking level. Somewhat counter intuitively, the cooperative coupling need not be present in the channels that are involved in the action potential generation (e.g. Na and K) but it seems that it is sufficient that cooperativity of some depolarising cationic channels in the membrane is present.

We thank the reviewer for this positive assessment and for referencing the Kim et al. report on cooperative gating of human potassium channels. For the revised article, we now cite this work in the Introduction and the Discussion.

1) Can the authors comment on whether the cooperativity of depolaring channels that are involved in the AP generation leads to the same or different net-effect (bistability) as that of other depolarising cationic channels. Is there any functional difference?

We expect that strong cooperativity between channels involved in the action potential (AP) generation is less suitable to mediate bistability between a silent and a firing state.

For example, consider that fast sodium channels were strongly cooperative and formed bistable clusters. Then, there is a danger that during an action potential the sodium channels become trapped in the open state and the neuron cannot emit further spikes. In principle, additional mechanisms like inactivation or large potassium currents could unblock the neuron by resetting the sodium channels. Such a reset, however, would also erase the memory.

Therefore, we think that for a functional bistability based on strong cooperativity, the coupling should occur in dedicated channels besides the AP-generating ones – for example a small population of calcium conductances as discussed in the manuscript. To emphasise this point, we address this question now in the discussion:

“Independent of the ion type, we expect that the cooperative channels are separate from the action potential-generating sodium and potassium channels, or at least form only a small subgroup therein. This separation would prevent memory from interfering with the action potential, the first based on persistent currents and the latter on regenerative, memoryless currents.”

2) How does the bistable switch operate in the presence of noise, are there any spontaneous switches? How does the duration of the upper state relate to the noise level?

We agree that the bistable clusters need to be robust against major noise sources like channel and voltage noise.

Experimentally, cluster sizes have been found to be relativity small – around ten channels per cluster – so that spontaneous gating events by individual channels present a dominant noise source. All our simulations incorporate this channel noise, e.g. in the voltage clamp simulations channels open and close randomly (see cluster traces in Figure 1C, Figure 2A and Figure 3A). Channel noise can destabilize both the open and closed state of a cluster and thereby limit its lifetime as a bistable switch. In the section “Prolonged life times of the open and closed cluster state”, we calculate these life times and show that a cluster can reside in a state for multiple seconds, which exceeds the life times of single channel states by multiple orders of magnitude. Cluster size effectively controls the level of channel noise; in larger clusters, channel noise becomes less prominent and spontaneous switches occur less often (see Figure 3B).

Thus, clusters are relatively robust against spontaneous switches and interestingly, they even rely on channel noise for their extended memory: the graded opening of clusters. If the cluster gating was deterministic, they would all open at the same time. Channel noise, in contrast, enables the observed graded response: each spike creates a window for channel noise to switch a few clusters to the upper branch (e.g. Figure 4A and C, Figure 5). Hence, channel noise effectively increases the memory capacity of the clusters.

The other noise source are voltage fluctuations, which stem from other ion channels in the membrane and fluctuating synaptic input. For example, in our dynamic clamp experiment, the recorded neurons exhibited intrinsic voltage fluctuations of 1 to 2 mV (synaptic transmission was blocked). The observed stable persistent activity already indicates that the clusters are robust against biological noise levels (see Figure 6). The robustness stems from the long cluster life times within the bistable voltage range (Figure 2A). Small fluctuations around the center of the bistable range therefore hardly affect the clusters.

For the revised manuscript, we performed additional simulations to show that clusters are also robust against stronger voltage fluctuations. We applied white noise current of different intensities to the model neuron and confirmed that spontaneous, noise-driven spiking at low frequencies cannot accidentally open clusters:

“Similarly, we find that the slow channels make the clusters robust against fluctuation-driven firing (Schreiber et al., 2009) – action potentials triggered by noise in the membrane potential (see Figure 4—figure supplement 2).”

3) From spiking networks we know that presence or absence of bistability can depend on the size of the network. The depolaring pulse to induce a switch needs to be stronger and longer for larger networks, ~to sqrt(N). Does the size of the cluster and the size of the switch inducing pulse correlate in some way? Can the authors comment on what a realistic regime could be?

Indeed, in the voltage clamp simulations discussed in the section “Hysteresis and bistable gating”, there is a correlation of cluster size and pulse amplitude required to switch the cluster. To switch the cluster, a pulse has to kick it out of the bistable voltage range, which for a fixed channel coupling *j* grows with increasing cluster size (see Figure 2C). As this relation of cluster size and pulse height nicely illustrates the importance of the bistable range, we decided to mention this fact in the section on the hysteresis and bistable gating:

“With increasing *J*, the bistable range broadens and extends to more hyperpolarized voltages (Figure 2C). Correspondingly, for a cluster with more channels and/or strong coupling, which both result in a larger *J*, the voltage pulse to close the cluster has to be stronger.”

As indicated above, the bistable range grows asymmetrically with different effects for hyper- and depolarizing pulses. Hyperpolarizing pulses, that close an open cluster, have to be stronger for larger clusters. They have to reach voltages below the lower edge of the bistable range, which decreases linearly with the cluster size S. In contrast, the upper edge remains roughly at the same voltage, so that depolarizing pulses do not need to change.

Why lower and upper edge of the bistable range behave so differently can intuitively be understood by looking at the S-shaped activation curve (Figure 1B). The upper edge is the point where the normal activation curve starts to “bend backwards”. It corresponds to the voltage where an individual channel starts to activate and is therefore more dependent on the form of the activation curve than on the coupling between channels. The lower edge, however, is the voltage where cooperative facilitation does not suffice to keep a channel open and therefore directly depends on coupling strength.

What is a realistic regime for cluster size and channel coupling? Moreno and colleagues found that clusters of cooperative calcium channels in hippocampal neurons contained up to 20 channels with a mean of 8 channels (Moreno, 2016, Figure 3F). In our channel model, a cluster with 8 channels, all-to-all coupled, requires a channel coupling *j* of about 5 mV to become bistable (see Figure 3B). For the simulations and the dynamic clamp on persistent activity, we chose a coupling of 10-15 mV (see Table 1). Coupling strengths have not yet been measured experimentally, but channel recordings of cooperative calcium channels in the heart seem to indicate that a small number of cluster could indeed be bistable (Navedo, 2010, see Figure 1E and F).

4) I think it would be a good idea to streamline the Introduction and Discussion and cut the number of figures and panels. It does seem like the last 3 figures have somewhat overlapping messages.

We appreciate the suggestion to make the manuscript more concise. Following your proposal, we have edited the Introduction and Discussion. Still, we think that a more detailed background on ion channel cooperativity is valuable to the community.

We agree that in the initial version of the manuscript Figures 4 and 5 had partially overlapping messages and could have been merged. In the revised version, however, we added supplementary figures to each one of them and feel that they address separate issues. For example, we showed that the cluster bistability can also be used to mediate hyperpolarization activated persistent activity (Figure 4—figure supplement 1). We think that this is an interesting aspect of the general memory mechanism enabled by the cluster bistability (Figure 4), but that it would fit less to the specific discussion of graded persistent activity (Figure 5).

Reviewer #2:

[…] The idea of coupling between ion channels as a form of memory is an elegant concept. I do wish the authors would take it a bit further: there are a number of channels that have demonstrated cooperative gating and more generally coupled gating. These vary widely from K currents to gap junctions with a variety of associated kinetics – fast versus slow gating, low versus high conductance, long versus short open times and latencies to opening etc. I think the authors could do a more expansive exploration of the potential memory dynamics that could be coded within such a large parameter space. Such an analysis may open more doors for future computational and experimental exploration.

We thank the reviewer for the positive assessment and appreciate the suggestion to further explore memory dynamics of clusters with respect to different single channel characteristics. To this end, we chose to concentrate on three basic channel properties: fast versus slow gating, low versus high conductance, and a change in reversal potential from a depolarizing to a hyperpolarizing channel. Although these parameters have no effect on the cluster bistability, they determine capacity and form of the neuron’s memory. For high capacity memory represented by multiple stable levels of persistent activity, the channels should gate slowly and have intermediate conductance levels (see Figure 5—figure supplement 1 added in the revised manuscript). Interestingly, the form of persistent activity changes when the cooperative channels conduct hyperpolarizing currents – from depolarization-activated to hyperpolarization-activated (see Figure 4—figure supplement 2 added in the revised manuscript). In our opinion, this additional exploration helps to understand the memory mechanism of the cooperative channels, so that in the revised manuscript, we refer to it both in the Results and in the Discussion.

Low versus high conductance: In a rerun of the graded persistent activity simulation, we varied the conductance around the original value of 2.5 pS per channel and monitored how the persistent activity level changed (see Figure 5—figure supplement 1). When the conductance is lowered to 0.5 pS, the persistent current is insufficient to drive spiking and the neuron stays silent. When the conductance is close to the original value, the neuron spikes persistently at increasing levels with each pulse. However, with even more conductive channels, 10 pS, the graded response is lost. With a higher conductance, clusters can drive firing at frequencies above 15 Hz, which will open further clusters and destabilize all levels except the highest frequency.

Fast versus slow channels: The time constant influences the life time of cluster states (see Figure 3) and the speed of their response to spikes (see section “Neural signaling events lead to controllable, persistent cluster switches” and Figure 4). To illustrate the effects on the memory dynamics, we repeated the graded persistent activity experiment, now varying the time constant (see Figure 5—figure supplement 1). As expected, the simulations show that if the channels are too fast, there is only one level of persistent activity. For fast channels <10 ms, a few spikes suffice to open all channels, which suppresses all intermediate levels.

Reversal potentials: Last, we investigated the effect of the reversal potential to demonstrate that mechanism of persistent activity is not idiosyncratic for one specific channel type (nor one specific ion type that is conducted). The added analysis allows us to understand how, for example, cooperative potassium channels could support neuronal memory. In this case, reducing a standing leak current through the potassium channels can drive spiking (Zylberberg, 2017 and Cui, 2018). Interestingly, the scenario of reducing a standing leak current is readily implemented with the cooperative channels and results in an interesting variant of persistent activity, which is activated by hyperpolarization and turned off by strong spiking (Figure 4—figure supplement 1). In short, when all cooperative potassium channels are open, they prevent the cell from firing. Strong hyperpolarization then switches the channels to the closed state, so that the standing leak current vanishes and the cell can fire. If the cell is stimulated to fire at high frequencies, the clusters reopen and silence the cell.

In future work, we plan to continue this exploration. A particularly interesting route is consider cooperative coupling between channels with more complex gating dynamics than the single-gated channel considered here.

Reviewer #3:

[…] 1) What turns off open channels in cooperative clusters? Say in Figure 1, at V=V_0.5_, activation curve for strongly cooperative channels (Figure 1B) is at 1. Why would all channels close at about same time? Is their closing (inactivation?) also cooperative?

A more comprehensive analysis of opening/closing dynamics of a cluster may help here.

It is clear that a strong hyperpolarization can turn channels off, but how would this happen spontaneously?

Thank you for putting forward this question about the closing of cooperative channels.

First, we apologize for a mislabeling of Figure 1, which we have now corrected. Specifically, in the legend of Figure 1 it should have read V=const, because (as indicated in the caption) the clamping voltages are different for independent and cooperative channels. The independent channels were clamped at -1 mV, the half activation V_0.5_ of the single channels, and the cooperative channels were clamped at -36 mV, the middle of their bistable range. We now have added the clamping voltages to every trace in panel C. For reference, we also marked them in panel B, so that it is easier to locate them with respect to the activation curves.

Second, why do open channels in a cooperative cluster close spontaneously and quasi-synchronously? It is indeed more intuitive that cooperativity enables a spontaneous, quasi-synchronous opening of channels in a cluster: each open channel further increases the activation of its neighbors and thereby triggers a fast cascade of opening events. To understand the synchronous closing, let us consider the situation where all channels are initially open. When one channel closes, all of its neighbors loose one open neighbor, so that they become more likely to close as well. In the same way, this loss of activation can perpetuate and induce a cascade of closings. At this point, we would like to emphasise that actually more than one channel has to open, respectively to close, in order to trigger such a cascade. This is the reason why the cluster is much more stable than the single channels (see Figure 3).

As we think that this question could be interesting for all readers, we have adapted our description of the cooperative gating dynamics:

“Intuitively, this coordination within a cluster can be easily understood: the opening of ion channels strongly enhances the probability of their neighbors to open. Similarly, the closing of channels reduces the probability of their neighbors to remain open, so that gating dynamics largely synchronize.”

Moreover, we added a supplementary figure to clarify how channel noise leads to spontaneous cluster switches:

“These switches originate in channel noise, stochastic gating of individual channels. If multiple channels coincide in their spontaneous gating, their neighbors quickly follow. Both opening and closing of channels spread by cooperative coupling; in a spiral of facilitation build-up or, reversely, in a spiral of facilitation loss (Figure 3—figure supplement 1).”

2) Conductance through permanently open channel clusters might change the membrane potential (e.g. in Figures 4 or 5 simulations). Certainly, resulting Vm shift would depend on the ratio of conductance through channel clusters to other conductances, but since it influences firing, it might also influence the Vm.

Indeed, the permanent opening of the clusters changes the membrane potential even when the cell does not fire yet. When only a few clusters are open, the additional conductance is small in relation to the leak, so that the membrane potential only increases slightly and the neuron does not spike: a form of “silent” memory. Eventually, another stimulation opens further channels, so that the membrane potential increases up to threshold and the neuron starts to fire.

As this “silent” memory might be of interest for other neuronal memories, like persistent changes in excitability, we now explicitly mention it in the Results section:

“Finally, the cell is slightly depolarized, so that it spikes in the absence of a stimulus when several clusters are open (see Figure 4C). Even when there are too few open clusters to induce spiking, they still increase the membrane potential and leave a form of “silent” memory (not shown).”

3) Writing is often in very general terms, lacking specific details; e.g.:

– Authors talk about "channels" – which channels? If specifics of ionic conductance does not matter, this should be clearly stated in the very beginning.

We have now edited the manuscript to make explicit, when the specifics of the ionic conductance matter. In this first part on the bistability of the clusters, the ion type conducted by the channels has no influence. We introduce them as “channels with simple and generic activation dynamics” and emphasize “that we make no further assumptions on other channel properties like their ionic nature and therefore simply refer to them as "channels"”. In the second part on persistent activity, the ion type becomes important. We first consider “that they conduct a depolarizing current like for example calcium channels”, but then also discuss which memory dynamics are possible with channels that “conduct a hyperpolarizing current like for example potassium channels”, see Figure 4—figure supplement 1). Finally, we added a short discussion on the ion type of the channels in the Discussion (subsection “Ion channel cooperativity as a mechanism for graded persistent activity”, last paragraph).

– Introduction (last paragraph), it is not clear that you are talking about model simulations.

Changed to “Here, we show in simulations and mathematical analysis that small clusters of cooperative channels […]”

– Materials and methods: description of electrophysiological experiments is very vague. Recording from which brain area? Which cells were targeted?

We added more details in the Materials and methods: “Single cell recordings were obtained from principal neurons of the perirhinal cortex layer II. These neurons show graded persistent firing under activation of muscarinic cholinergic receptors (Navaroli, 2012).”
